# Beta-blockers for the prevention of headache in adults, a systematic review and meta-analysis

**DOI:** 10.1371/journal.pone.0212785

**Published:** 2019-03-20

**Authors:** Jeffrey L. Jackson, Akira Kuriyama, Yachiyo Kuwatsuka, Sarah Nickoloff, Derek Storch, Wilkins Jackson, Zhi-Jiang Zhang, Yasuaki Hayashino

**Affiliations:** 1 Department of Medicine, Zablocki VA Medical Center, Milwaukee, WI, United States of America; 2 Department of General Medicine, Kurashiki Central Hospital, Okayama, Japan; 3 Department of Medicine, Nagoya University Hospital, Nagoya, Japan; 4 Department of Biology, University of Wisconsin-Milwaukee, Milwaukee, WI, United States of America; 5 Department of Epidemiology and Biostatistics, School of Health Sciences, Wuhan University, Wuhan, China; 6 Department of Endocrinology, Tenri Hospital, Nara, Japan; Keele University, UNITED KINGDOM

## Abstract

**Background:**

Headaches are a common source of pain and suffering. The study’s purpose was to assess beta-blockers efficacy in preventing migraine and tension-type headache.

**Methods:**

Cochrane Register of Controlled Trials; MEDLINE; EMBASE; ISI Web of Science, clinical trial registries, CNKI, Wanfang and CQVIP were searched through 21 August 2018, for randomized trials in which at least one comparison was a beta-blocker for the prevention of migraine or tension-type headache in adults. The primary outcome, headache frequency per month, was extracted in duplicate and pooled using random effects models.

**Data synthesis:**

This study included 108 randomized controlled trials, 50 placebo-controlled and 58 comparative effectiveness trials. Compared to placebo, propranolol reduced episodic migraine headaches by 1.5 headaches/month at 8 weeks (95% CI: -2.3 to -0.65) and was more likely to reduce headaches by 50% (RR: 1.4, 95% CI: 1.1–1.7). Trial Sequential Analysis (TSA) found that these outcomes were unlikely to be due to a Type I error. A network analysis suggested that beta-blocker’s benefit for episodic migraines may be a class effect. Trials comparing beta-blockers to other interventions were largely single, underpowered trials. Propranolol was comparable to other medications known to be effective including flunarizine, topiramate and valproate. For chronic migraine, propranolol was more likely to reduce headaches by at least 50% (RR: 2.0, 95% CI: 1.0–4.3). There was only one trial of beta-blockers for tension-type headache.

**Conclusions:**

There is high quality evidence that propranolol is better than placebo for episodic migraine headache. Other comparisons were underpowered, rated as low-quality based on only including single trials, making definitive conclusions about comparative effectiveness impossible. There were few trials examining beta-blocker effectiveness for chronic migraine or tension-type headache though there was limited evidence of benefit.

**Registration:**

Prospero (ID: CRD42017050335).

## Introduction

Headaches are a common problem, world-wide. The two most common types of headaches are migraine and tension-type. Migraines have a prevalence of 6–8% [1–9], and cause significant disability [10–13], even during periods between attacks [14]. Migraines are responsible for $1 billion in medical costs and $16 billion in lost productivity per year [15;16] in the US alone. While episodic migraine is more common than chronic migraine, chronic migraine has greater disability as well as financial and occupational consequences [8;9] and has received much greater research attention [17].

Tension-type headache is more common than migraine; up to 90% of adults experience one at some time in their life [18–22]. In any given month, a tension-type headache occurs in 46% of adults [22]. Most tension-type headaches are managed with over the counter medications, consequently most do not seek medical attention. However, tension-type headache reduces the quality of life [23], results in up to a fifth of all missed work days [24], and costs EUR 21 billion annually in Europe [25].

There are several options available for preventing migraines including alpha antagonists, antiepileptics [26], beta-blockers [27], botulinum toxin-A [28], calcium channel blockers [29], flunarizine [17], pizotifen [17], serotonin agonists [30], serotonin reuptake inhibitors (SSRIs) [31] and tricyclic antidepressants (TCAs) [32]. Nearly half of males and a third of females who are candidates for prophylactic therapy do not receive it [33]. Selection of prophylactic treatment is tailored on individual patient characteristics, costs, perceived efficacy of the intervention and side effects of the available options.

The 2012 American Academy of Neurology guideline recommends beta-blockers, specifically propranolol and metoprolol, as first line therapy for preventing migraines [34]. Specific medications commonly used in prophylaxis has not been well described. In Europe, commonly prescribed prophylactic agents include antiepileptics, beta-blockers, flunarizine, pizotifen and TCAs [35]. Other studies found that specialists are twice as likely to prescribe antiepileptics than primary care providers [36], that treatment persistence is low [37] and that use of prophylactic medications has increased [38], though none of these three characterized the specific medications used.

The purpose of this study is to assess the efficacy of beta-blockers in the prophylaxis of migraine and tension-type headache. Two previous systematic reviews focused on the use of beta-blockers in migraine headaches, both are more than 15 years old [39;40], and included limited outcomes, though both suggest benefit of beta-blockers compared to placebo. There are two more recent comparative effectiveness analyses of headache management that included beta-blockers. Shamliyan reviewed pharmacologic treatment for episodic migraine and reported that beta-blockers were effective; their outcome was 50% reduction in headaches, an outcome recommended by the International Headache Society (IHS) as a secondary outcome. They also excluded beta-blockers not approved for headaches in the U.S [41]. In the other meta-analysis, we found that beta-blockers were beneficial for migraine headaches, but did not differentiate between episodic and chronic migraine headaches, did not include all possible outcomes and did not examine beta-blockers for management of tension-type headache [17].

## Methods

This study was conducted in accordance with PRISMA guidelines (S1 Table. Prisma Checklist) [42] and was registered in PROSPERO (ID: CRD42017050335). Databases searched (without language restriction) included the Cochrane Central Register of Controlled Clinical Trials, MEDLINE, EMBASE, ISI Web of Science (SCI, SSCI, CPCI-S & CPCI-SSH), and three Chinese databases (CNKI, Wanfang and CQVIP) trough 21 August 2018 using the search strategies in supporting information (S2 Table. Search Strategy). Randomized controlled studies of adults that were at least four weeks in duration and used a beta-blocker in at least one study arm were included. Articles were assessed for inclusion in duplicate (JLJ, AK). Because the definition of headache has changed over time, articles were reviewed by at least two authors to determine if the headache could be reasonably classified as migraine or tension-type headache and as either frequent episodic or chronic according to the most recent IHS criteria [43]. IHS recommendations were followed by including only patient-reported outcomes [44] and including the monthly headache frequency as the primary outcome. Additional outcomes included headache index, headache days, severity, duration, quality of life, the use of acute analgesic medications, the proportion with at least 50% improvement in headaches, study withdrawal and the occurrence of adverse events. Data were abstracted in duplicate. Because of the large volume of articles, after training on a separate set of pediatric headache articles, the articles were divided among the authors with all authors serving as the primary abstractor for some articles and as the secondary reviewer, assessing for data accuracy in other articles. Disagreements resolved through consensus between the two and if consensus could not be reached, the entire group discussed and made consensual decisions.

Bias was assessed using the Cochrane risk of bias instrument [45] as well as the JADAD scale [46]. Study size was also included as a risk of bias based on sample size calculations. It was estimated that 60 subjects were required for continuous outcomes and 200 for dichotomous ones (S3 Table. Quality Ratings of Included Trials) based on results from our previous review of treatment of migraine headaches [17]. Studies with more than one arm were pooled into a single arm (if the study reported no differences between arms). For crossover trials, several approaches were used, depending on how the data was reported. For trials that provided only pooled data from both time-periods, the sample size was reduced by 50%, to avoid over-weighting the study [45]. For trials that provided data from both time periods separately, if there was no difference between the two-time periods, the average point estimates and variance was used, with reduction of the sample size by 50%.

The preference was to pool study outcomes in their original unit of analysis. Headache frequency was pooled as headache days per month, headache duration as hours per month, and analgesic use as number of doses per month. Since headache severity and headache index metrics varied, these outcomes were pooled using standardized mean differences [47]. Missing outcome variances were imputed from the reported mean, sample size, and P values [48]. Heterogeneity was assessed using Chi^2^ (Cochrane Q), Galbraith plots [49] and the I^2^ statistic [50]. Data were pooled at each reported time point using a random-effects model [51] using Stata (V15.1 College Station TX). A priori, the analytic plan was to pool data at 4, 8, 12, 16, 20 and 24 weeks. Studies that reported outcomes at different time points were combined at the closest time point available (for example, 9-week outcomes were pooled with the 8-week group). For comparative effectiveness trials with 2 or fewer studies, outcomes were reported at the last point reported. Small study effects (publication bias) was assessed using the methods of Peters [52] for dichotomous outcomes and Egger [53] for continuous outcomes if there were a sufficient number of studies. Trial sequential analysis was performed for the comparisons of propranolol to placebo for headache frequency (at 8 weeks), using the O’Brien-Fleming method of alpha-spending function to robustness of the pooled estimates against type 1 and type 2 error [54], using TSA software (Copenhagen, Denmark).

A network meta-analysis was performed for beta-blockers that were compared with placebo at 8 and 12 weeks using the residual maximum likelihood with a modification to the coefficients’ estimated variance using the Kapp and Hartung approach [55] that had a minimum of 2 studies. Both 8- and 12-week results were pooled including all beta-blockers using multivariate random-effects meta-analysis using the network package in STATA [56].

Finally, the quality of evidence was assessed using the GRADE (Grades of Recommendation, Assessment, Development and Evaluation) system to rate the quality of the evidence (GRADEPro GDT 2015) following Cochrane guidelines [45]. Grade assesses quality in four levels: High (further research is unlikely to change estimate of effect); Moderate (further research may impact effect estimate); Low (further research is likely to have important impact on estimate); Very low (any estimate of effect is very uncertain).

## Results

**Fig 1:**
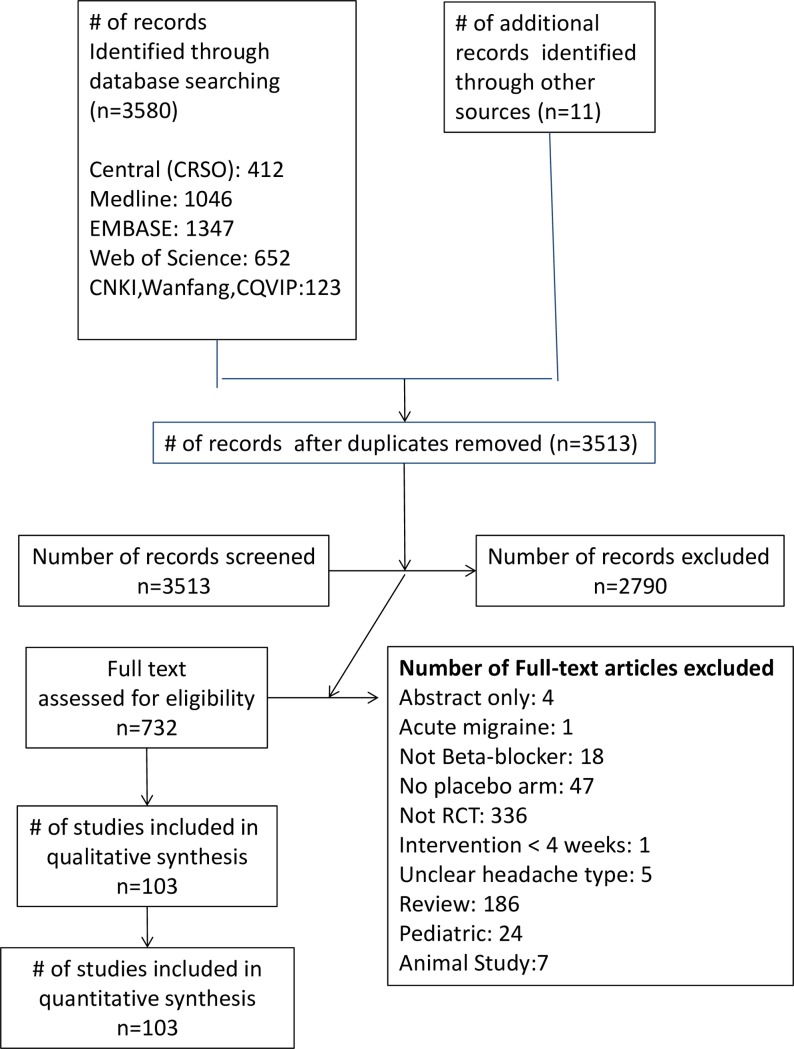
PRISMA flow chart.

The literature search yielded 3513 unique studies after excluding duplicates. Application of inclusion and exclusion criteria (Fig 1) resulted in 108 randomized controlled trials [57–164], of which 50 had a placebo arm [57–59;62;68;69;71;72;75;77–79;81–83;88;94–97;99;107;108;113;115;116;118;121–124;126;130;133–138;141;142;144;145;147;149;151;152;152;154;156;157;162]. Because some placebo-controlled trials included non-placebo comparisons, there were a total of 86 comparative effectiveness arms. Nearly all trials (n = 106) focused on migraine headaches, most (n = 83) could be classified as episodic. Only 4 trials studied chronic migraines [76;105;129;151] and there was only 1 trial of beta-blockers for chronic tension-type headache [57].

The 108 included studies ranged from 4 to 64 weeks in duration (average: 12.9). Fifty-one were parallel in design and 57 had a crossover design. Among crossover trials, 43 were randomized, with washout periods ranging from zero to four weeks. Twenty-five different countries (Table 1) and four languages (Chinese (n = 15), English (n = 86), German (n = 6), Polish (n = 1) were represented. The average age was 38.6 years and 77% of participants were women. Ten different beta-blockers were studied. Propranolol (n = 74) and metoprolol (n = 21) were the most commonly evaluated beta-blockers. Atenolol, nadolol, pindolol and timolol had two studies each. Several beta-blockers were evaluated in only a single trial (acebutolol, alprenolol, bisoprolol and oxprenolol). Study characteristics for included trials are provided in Table 1 and quality ratings are given in S2 Table.

**Table 1:** Included trial characteristics.

Study, Year, Country	Comparison (mg)	Headache Type	Classification	Design	Duration (weeks)	Sample Size (dropout %)	Age	Women	Dose titrated	Rescue Medication Allowed
**Acebutolol**										
Nanda, 1978, Scotland	Acebutolol (800) v. Placebo	Migraine-episodic	NS	Crossover	12	43 (23%)	NS	74%	Yes	Yes
**Alprenolol**										
Ekbom, 1975, Sweden	Alprenolol (200) v. Placebo	Migraine-episodic	Ad-hoc 1962	Crossover	6	33 (15)	41.5	82%	No	Yes
**Atenolol**										
Forssman, 1983, Sweden	Atenolol (100) v. Placebo	Migraine-unspecified	Ad-hoc 1962	Crossover	13	24 (17)	40	80%	No	Yes
Johannsson, 1987, Sweden	Atenolol (100) v. Placebo	Migraine-episodic	Ad-hoc 1962	Crossover	12	72 (13)	43	70%	No	Yes
Stensrud, 1980, Norway	Propranolol (160) v. Atenolol (100)	Migraine-episodic	Ad-hoc 1962	Crossover	6	35 (20)	NS	69%	No	Yes
**Bisoprolol**										
van de Ven, 1997, Europe	Bisoprolol (5) v. Bisoprolol (10) v. Placebo	Migraine-episodic	IHS1988	Parallel	12	226 (14)	38.7	82%	No	Yes
**Metoprolol**										
Andersson, 1983, Denmark	Metoprolol (200) v. Placebo	Migraine-episodic	WFNRG 1969	Parallel	8	71 (13)	39.7	85%	No	Yes
Diener, 2001, Europe	Metoprolol (200) v. Aspirin (300)	Migraine-episodic	IHS 1988	Parallel	16	270 (15)	39.4	81%	Yes	Yes
Gong, 2016, China	Metoprolol (25) + Flunarizine (5) v. Flunarizine (5)	Migraine-unspecified		Parallel	12	80 (0)	47.5	40%	No	Yes
Grotemeyer, 1988, Germany	Metoprolol (200) v. Flunarizine (10)	Migraine-episodic	Ad hoc 1962	Crossover	7	29 (17)	39	79%	No	Yes
Grotemeyer, 1990, Germany	Metoprolol (200) v. Acetylsalicyclic Acid (1500)	Migraine-episodic	IHS 1988	Crossover	12	28 (NS)	31	82%	No	Yes
Hesse, 1994, Denmark	Metoprolol (100) v. Acupuncture	Migraine-episodic	IHS 1988	Parallel	17	85 (10)	44.7	84%	No	Yes
Kangasniemi, 1987, Scandinavia	Metoprolol (200) v. Placebo	Migraine-episodic	Ad-hoc 1962	Crossover	8	77 (11)	37.5	80%	No	Yes
Langohr, 1985, Germany	Metoprolol (100) v. Clomipramine (100) v. Placebo	Migraine-episodic	Ad-hoc 1962	Crossover	4	63 (43)	44.4	67%	No	Yes
Li, 2006, China	Metoprolol (125) v. Placebo	Migraine-unspecified	IHS 1988	Parallel	12	60 (0)	48.5	100%	No	Yes
Louis 1985, Europe	Metoprolol (100) v. Clonidine (0.1)	Migraine-episodic	WFNRG 1969	Crossover	8	31 (26)	35.5	81%	Yes	Yes
Ma, 2011, China	Metoprolol (50) + Flunarizine (5) v. Flunarizine (5)	Migraine-episodic	HIS 2004	Parallel	48	56 (0)	36.3	65%	No	Yes
Schellenberg, 2008, Germany	Metoprolol (142.5) v. Nebivolol (5)	Migraine-episodic	IHS 2004	Parallel	18	30 (7)	39	87%	Yes	Yes
Siniatchkin, 2007, Germany	Metoprolol (200) v. Placebo	Migraine-unspecified	IHS2004	Parallel	12	20 (0%)	37	85%	Yes	Yes
Sorensen 1991, Denmark	Metoprolol (200) v. Flunarizine (10)	Migraine	IHS 1988	Parallel	20	149 (1)	42	79%	No	Yes
Steiner, 1988, UK	Metoprolol Cr (100) v. Placebo	Migraine-episodic	Vahlquist 1955	Parallel	8	59 (NS)	37.4	76%	No	Yes
Streng 2005, Germany	Metoprolol (200) v. Acupuncture)	Migraine-episodic	IHS 1997	Parallel	12	114 (17)	36.6	88%	Yes	Yes
Vilming, 1985, Sweden	Metoprolol (100) v. Pizotifen (1.5)	Migraine-episodic	WFNRG 1969	Crossover	8	35 (14)	37.6	83%	Yes	Yes
Worz 1992, Germany	Metoprolol (200) v. Bisoprolol (10)	Migraine-episodic	IHS1988	Crossover	12	125 (38)	38.5	71%	Yes	Yes
Yang, 2006, China	Metoprolol (90) v. Placebo	Migraine-episodic	IHS 1988	Parallel	12	60 (0)	48.5	100%	No	Yes
Yang, 2016, China	Metoprolol (95) v. Metoprolol (95) + Fluoxetine	Migraine-episodic	NS	Parallel	6	120 (0)	38.4	64%	No	Yes
Zhou, 2015, China	Metoprolol (95) v. Metoprolol (95) + Fluoxetine	Migraine-episodic	NS	Parallel	6	112 (0)	37.0	63%	No	Yes
**Nadolol**										
Freitag, 1984, USA	Nadolol (80) v. Nadolol (160) v. Placebo	Migraine-unspecified	Ad-hoc 1962	Parallel	12	32 (20)	36.7	81%	No	Yes
Ryan, 1982, USA	Nadolol (80) v. Nadolol (160) v Nadolol (240) v Placebo	Migraine-episodic	NS	Parallel	12	80 (1%)	NS	78%	No	Yes
**Oxprenolol**										
Ekbom, 1977, Sweden	Oxprenolol (240) v. Placebo	Migraine-episodic	Ad-hoc 1962	Crossover	12	34 (12)	41.8	76%	No	Yes
**Pindolol**										
Ekbom, 1972, Sweden	Pindolol (7.5) v. Pindolol (15) v. Placebo	Migraine-episodic	Ad-hoc 1962	Parallel	4	30 (NS)	33.7	87%	No	Yes
**Pindolol + Amitriptyline**										
Agius, 2013, Italy	Pindolol (10)+ Amitriptyline (10) v. Amitriptyline (10) v. Placebo	Tension-chronic	IHS 2004	Parallel	8	64 (3)	35.6	74%	No	Yes
Streng, 2005, Germany	Metoprolol (200) v. Acupuncture	Migraine-episodic	IHS 1997	Parallel	12	114 (17)	36.6	88%	Yes	Yes
**Propranolol**										
Ahuja, 1985, India	Propranolol (120) v. Placebo	Migraine-episodic	Ad-hoc 1962	Crossover	8	26 (NS)	NS	46%	No	NS
al-Qassab, 1993, UK	Propranolol (80) v. Propranolol (160) v. Placebo	Migraine-episodic	Ad-hoc 1962	Crossover	8	45 (33)	36	80%	No	Yes
Albers, 1989, USA	Propranolol (180) v. Nifedipine (60)	Migraine-episodic	Ad hoc 1962	Parallel	24	40 (37)	35.2	89%	Yes	Yes
Andersson, 1981, Denmark	Propranolol (160) v. Femoxitine (400)	Migraine	NS	Crossover	24	49 (24)	38	69%	Yes	Yes
Ashtari, 2008, Iran	Propranolol (80) vs. Topiramate (50)	Migraine-episodic	IHS 2005	Parallel	8	62 (3)	30.8	82%	Yes	Yes
Baldrati, 1983, Italy	Propranolol (80) v. Aspirin (1.9 mg/kg)	Migraine-not specified	Ad hoc 1962	Crossover	12	18 (33)	33.3	89%	No	NS
Behan, 1980, Scotland	Propranolol (120) v. Methysergide (3)	Migraine-not specified	NS	Crossover	12	56 (36)	NS	66%	No	No
Bonuso, 1998, Italy	Propranolol (80) v. Flunarizine (10)	Migraine-not specified	IHS 1988	Parallel	8	50 (16)	32	68%	No	NS
Bordini, 1997, Brazil	Propranolol (60) v. Flunarizine (10) v. Combo.	Migraine-episodic	IHS 1988	Parallel	17	52 (13)	31.2	91%	No	Yes
Borgesen, 1974, Denmark	Propranolol (120) v. Placebo	Migraine-episodic	Ad-hoc 1962	Crossover	12	12 (33)	37.6	83%	Yes	Yes
Carroll, 1990, UK	Propranolol (80) v. Propranolol (160)	Migraine-episodic	Ad hoc 1962	Crossover	12	51 (27)	39	69%	No	Yes
Chen, 2009, China	Propranolol (60) + Flunarizine (10) v. Topoiramate (100)	Migraine-episodic	IHS 1988	Parallel	12	82 (0)	38.2	60%	Yes	Yes
Dahlof, 1987, Sweden	Propranolol (120) v. Placebo	Migraine-episodic	WFNRG 1969	Crossover	4	28 (0)	NS	93%	No	Yes
Diener, 1996, German	Propranolol (120) v. Cylcendalate (1200) v. Placebo	Migraine-episodic	IHS 1988	Parallel	12	214 (17)	39	78%	Yes	Yes
Diener, 2002, Germany	Propranolol (160) v. Flunarizine (5) v. Flunarizine (10)	Migraine-episodic	IHS 1988	Parallel	16	808 (18)	38.8	63%	Yes	Yes
Diener, 2004, Europe	Propranolol (160) v. Topiramate (100) v. Topiramate (200) v. Placebo	Migraine-episodic	IHS 1988	Parallel	26	575 (37)	41	80%	Yes	Yes
Domingues, 2009, Brazil	Propranolol (80) v. Nortriptyline (40) v. Combo.	Migraine-chronic	IHS 2004	Parallel	12	76 (42)	NS	NS	Yes	Yes
DongXiang, 2010, China	Propranolol (90) + Amitriptyline (100) v. Amitriptyline (100)	Migraine-episodic	HIS 1988	Parallel	12	310 (0)	32.5	80%	Yes	Yes
Formisano, 1991, Italy	Propranolol (120) v. Nimodipine (120)	Migraine-episodic	IHS 1988	Parallel	16	22 (14)	39.2	55%	No	Yes
Forssman, 1976, Sweden	Propranolol (240) v. Placebo	Migraine-unspecified	NS	Crossover	10	40 (20)	37.4	88%	No	Yes
Gawel, 1992, Canada	Propranolol (120) v. Flunarizine (10)	Migraine-episodic	WFNRG 1970	Parallel	16	94 (19)	35.9	89%	Yes	Yes
Gerber, 1991, Germany	Propranolol (120) v. Metoprolol (150) v. Nifedipine (30)	Migraine-episodic	IHS 1988	Parallel	12	58 (NS)	42.4	73%	Yes	Yes
Gerber, 1995, Germany	Propranolol (120/160) v. Cyclandelate (1200/1600)	Migraine-episodic	IHS 1988	Parallel	8	84 (26)	40.9	90%	No	Yes
Ghobadi, 2013, Iran	Propranolol (120) v. Nimodipine (30)	Migraine	IHS 2004	Parallel	24	102 (2)	47	83%	No	Yes
Grotemeyer, 1987, German	Propranolol (120) v. Placebo	Migraine-episodic	Ad-hoc 1962	Crossover	12	30 (20)	36	73%	No	Yes
Havanka-Kannianen, 1988, Finland	Propranolol (80) v. Propranolol (160)	Migraine-episodic	Ad-hoc 1962	Crossover	12	48 (13)	37.7	81%	No	Yes
Hedman, 1986, Denmark	Propranolol (80) v. Metoprolol (100)	Migraine-episodic	WFNRG 1970	Crossover	4	12 (0)	40	67%	NS	Yes
Holdorff, 1977, Germany	Propranolol (120) v. Placebo	Migraine-episodic	Ad-hoc 1962	Parallel	12	53 (30)	NS	NS	No	Yes
Holroyd, 2010, USA	Propranolol/Nadolol v. Propranolol/Nadolol + Behavior Therapy v. Behavior therapy v. Placebo	Migraine-episodic	IHS 1988	Parallel	64	232 (51)	38.2	79%	Yes	Yes
Jin, 2001, China	Propranolol (30) + Flunarizine (10) v. Diazepam (30) + Nimodipine (60)	Migraine	NS	Parallel	24	84 (0)	NS	75%	No	Yes
Johnson, 1986, New Zealand	Propranolol (240) Mefenamic Acid (1500) v. Placebo	Migraine-episodic	NS	Crossover	12	29 (41)	42	69%	No	Yes
Kangasniemi 1983, Finland	Propranolol (160) v. Femoxetine (400)	Migraine-episodic	NS	Crossover	12	29 (11)	37	86%	No	Yes
Kangasniemi 1984, Finland	Propranolol (240) v. Metoprolol (200)	Migraine-episodic	WFNRG 1970	Crossover	8	36 (6)	33.8	89%	No	Yes
Kaniecki, 1997, USA	Propranolol (240) v. Divalproex (1500)	Migraine-episodic	IHS 1988	Crossover	12	37 (14)	NS	81%	Yes	Yes
Kass, 1980, Norway	Propranolol (160) v. Clonidine (0.1)	Migraine-unspecified	WFNRG 1970	Crossover	16	23 (9)	39.7	30%	No	Yes
Kaushik, 2005, India	Propranolol (80) v. Biofeedback	Migraine-episodic	IHS 1988	Parallel	24	192 (13)	NS	69%	No	Yes
Ke, 2003, China	Propranolol (30) v. Propranolol (30) + Flunarizine (5) v. Flunarizine (5)	Migraine-chronic	IHS 1988	Parallel	8	121 (0)	31	74%	No	Yes
Kjaersgard 1994, Denmark	Propranolol (120) v. Tolfenamic Acid (300)	Migraine-unspecified	IHS 1988	Crossover	12	76 (26)	43.3	79%	No	Yes
Klapper, 1994,USA	Propranolol (140) v. Divalproex (1100)	Migraine-unspecified	IHS 1988	Crossover	8	24 (50)	NS	NS	Yes	Unclear
Kozubski, 1995, Poland	Propranolol (160) Valproaic Acid (1500)	Migraine-unspecified	IHS 1988	Crossover	10	35 (NS)	NS	100%	Yes	NS
Kuritzky, 1987, Israel	Propranolol (160) v. Placebo	Migraine-episodic	NS	Crossover	8	38 (18)	NS	NS	No	Yes
Li, 2002, China	Propranolol (30) v. Flunarizine (5)	Migraine-Episodic	IHS, 1988	Parallel	4	126 (0)	38.7	60%	No	Yes
Li, 2004, China	Propranolol (60) v. Valproate (.45 mg/kg)	Migraine-Episodic	NS	Parallel	36	40 (0)	NS	NS	No	NS
Lucking, 1988, Germany	Propranolol (120) v. Flunarizine (10)	Migraine-episodic	NS	Parallel	16	521 (NS)	42	80%	No	Yes
Maissen, 1991, Germany	Propranolol (120) v. 5-Hydroxytryptophan (300)	Migraine-episodic	NS	Parallel	16	39 (18)	39.4	67%	Yes	Yes
Malvea, 1973, USA	Propranolol (NS) v. Placebo	Migraine-episodic	NS	Crossover	6	31 (6)	NS	87%	Yes	Yes
Mathew, 1980, USA	Propranolol (160) v. Placebo v. Amitriptyline (75) v. Biofedback	Mixed- headaches	NS	Parallel	24	340 (20)	35.5	94%	Yes	Yes
Mikkelsen, 1986, Denmark	Propranolol (120) v. Tolfenamic Acid (300) v. Placebo	Migraine-episodic	Ad-hoc 1962	Crossover	12	31 (21)	39.4	84%	No	Yes
Nadelmann, 1986, USA	Propranolol (320) v. Placebo	Migraine-unspecified	Ad-hoc 1962	Crossover	12	57 (39)	NS	86%	No	Yes
Nair, 1975, India	Propranolol (80) v. Placebo	Migraine-episodic	NS	Crossover	8	20 (0)	27.3	50%	No	No
Nambiar, 2011, India	Propranolol (80) v. Riboflavin (100)	Migraine-episodic	IHS 1988	Parallel	24	100 (NS)	31	55%	Yes	Yes
Palferman, 1983, UK	Propranolol (120) v. Placebo	Migraine-unspecified	NS	Crossover	8	10 (38)	41.4	80%	No	Yes
Olerud, 1986, Sweden	Propranolol (80) v. Nadolol (80)	Migraine-episodic	NS	Parallel	24	28(NS)	NS	79%	No	Yes
Pita, 1977, Spain	Propranolol (160) v. Placebo	Migraine-episodic	Ad-hoc 1962	Crossover	8	9 (0)	32	78%	No	Yes
Pradalier, 1989, Norway	Propranolol (160) v. Placebo	Migraine-episodic	IHS 1988	Parallel	12	74 (26)	37.4	76%	No	NS
Ryan, 1984, USA	Propranolol (160) v. Nadolol (80) v. Nadolol (160)	Migraine-episodic	NS	Parallel	12	48 (6)	NS	73%	No	Yes
Sargent, 1985, USA	Propranolol (120) v. Naproxen (1100) v. Placebo	Migraine-episodic	NS	Parallel	14	149 (16)	30	79%	Yes	Yes
Shimell, 1990, South Africa	Propranolol (180) v. Flunarizine (10)	Migraine-episodic	Ad hoc 1962	Parallel	16	58 (2)	34	70%	Yes	NS
Silberstein, 2012, USA	Propranolol (240) + Topiramate (100) v. Topiramate (100)	Migraine-chronic	IHS 2006	Parallel	24	191 (39)	42	90%	Yes	Yes
Sjaastad, 1972, Norway	Pindolol (15) v. Placebo	Migraine-episodic	NS	Crossover	4	28 (14)	35.3	79%	Yes	Yes
Soyka, 1990, Germany	Propranolol (120) v. Flunarizine (10)	Migraine-unspecified	NS	Parallel	16	434	42	82%	Yes	Yes
Standnes, 1982, Norway	Propranolol (80) v. Timolol (10) v. Placebo	Migraine-episodic	Ad-hoc 1962	Crossover	12	25 (28)	41.4	80%	Yes	Yes
Stensrud, 1976, Norway	Propranolol (160) v. Placebo	Migraine-episodic	Ad-hoc 1962	Crossover	4	20 (5)	43.5	70%	No	Yes
Stensrud, 1980, Norway	Propranolol (80) v. Atenolol (50) v. Placebo	Migraine-episodic	Ad-hoc 1962	Crossover	6	35 (20)	NS	69%	No	Yes
Stovner, 2014, Norway	Propranolol (160) v. Candesartan (16) v. Placebo	Migraine-episodic	NS	Crossover	12	72 (15)	37	82%	Yes	Yes
Sudilovsky, 1987, USA	Propranolol (160) v. Nadolol (80) v. Nadolol (160)	Migraine-episodic	Ad hoc 1962	Parallel	12	140 (30)	39.3	76%	Yes	Yes
Tfelt-Hansen, 1984, Scandinavia	Propranolol (160) v. Timolol (20) v. Placebo	Migraine-episodic	Ad-hoc 1962	Crossover	12	96 (28)	39.5	74%	No	Yes
Weber, 1971, USA	Propranolol (20) v. Placebo	Migraine-unspecified	Ad-hoc 1962	Crossover	12	25 (24)	40.6	52%	No	Yes
Wideroe, 1974, Norway	Propranolol (160) v. Placebo	Migraine-episodic	Ad-hoc 1962	Crossover	12	30 (13)	40	90%	No	Yes
Wen, 2016, China	Propranolol (30) v. Flunarizine (10)	Migraine Episodic	NS	Parallel	8	100 (0)	25.6	65%	No	Yes
Yuan, 2005, China	Propranolol (120) v. Topiramate (150)	Migraine-unspecified	IHS 1988	Parallel	12	67 (0)	29.9	64%	Yes	No
Zhu, 2005, China	Propranolol (30) v. Flunarizine (10)	Migraine-unspecified	IHS 1988	Parallel	8	90 (0)	28.1	73%	No	No
Ziegler, 1993, USA	Propranolol (240) v. Amitriptyline (150) v. Placebo	Migraine-episodic	NS	Crossover	10	54 (44)	38	73%	Yes	Yes
**Timolol**										
Briggs, 1979, UK	Timolol (20) v. Placebo	Migraine-episodic	Ad-hoc 1962	Crossover	6	14 (7)	NS	71%	No	Yes
Stellar, 1984, USA	Timolol (30) v. Placebo	Migraine-episodic	Ad-hoc 1962	Crossover	8	107 (12)	43	72%	No	Yes

NS: Not Stated

Studies had a number of common quality problems (S3 Table) including high drop-out rates (16.1%, range 0–51%), lack of intention to treat analysis (76%), inadequate sequence generation (83%), lack of evidence of concealed allocation (90%) and inadequate blinding (60%). Twenty-three studies assessed compliance (21%). Fifty-one (47%) studies reported all collected outcomes. Sixteen trials (15%) were sponsored by industry. All comparisons that had only a single study were graded as low-quality evidence.

### Episodic migraines

**Fig 2:**
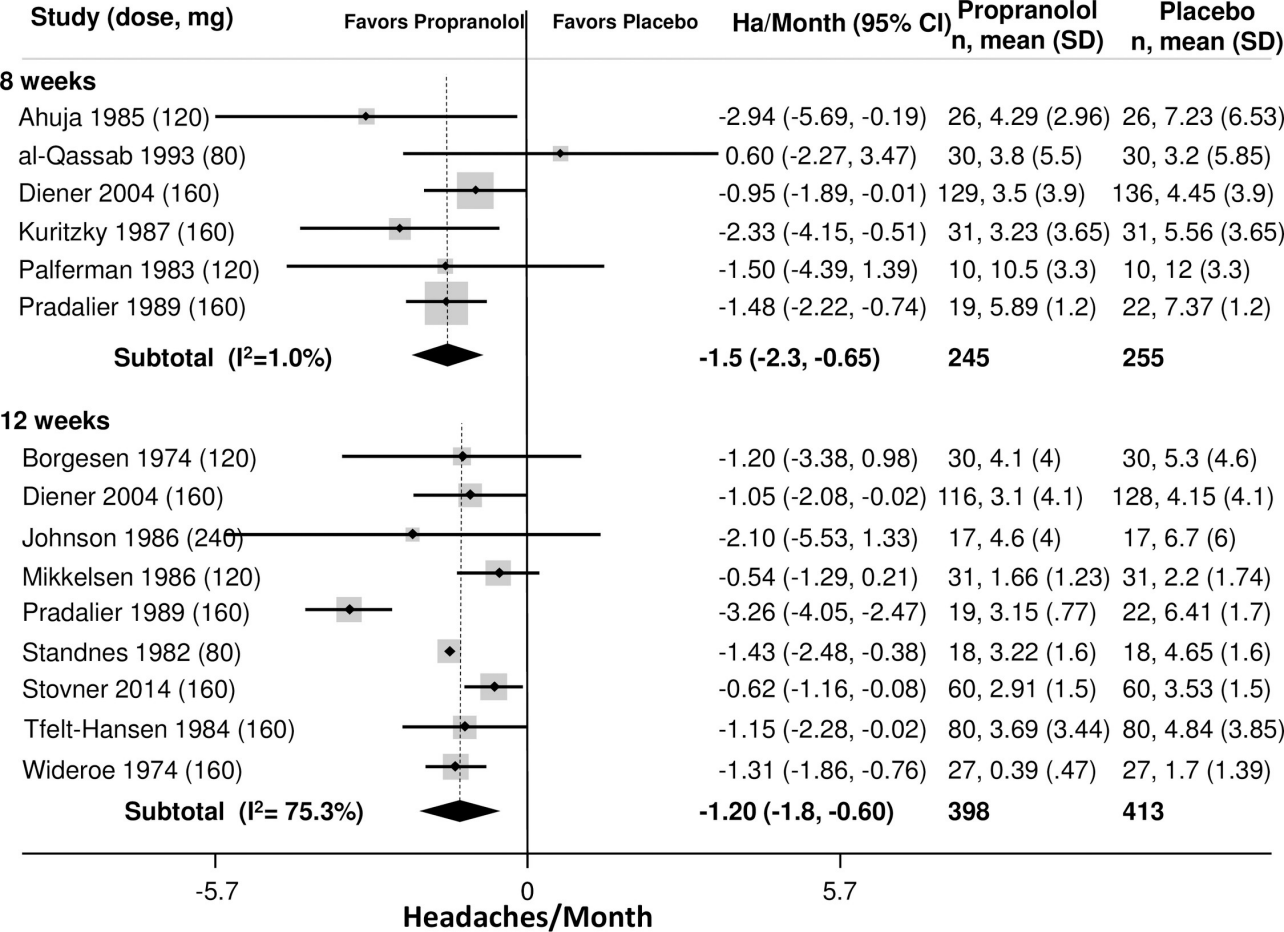
Headaches per month, Propranolol v. Placebo.

**Fig 3:**
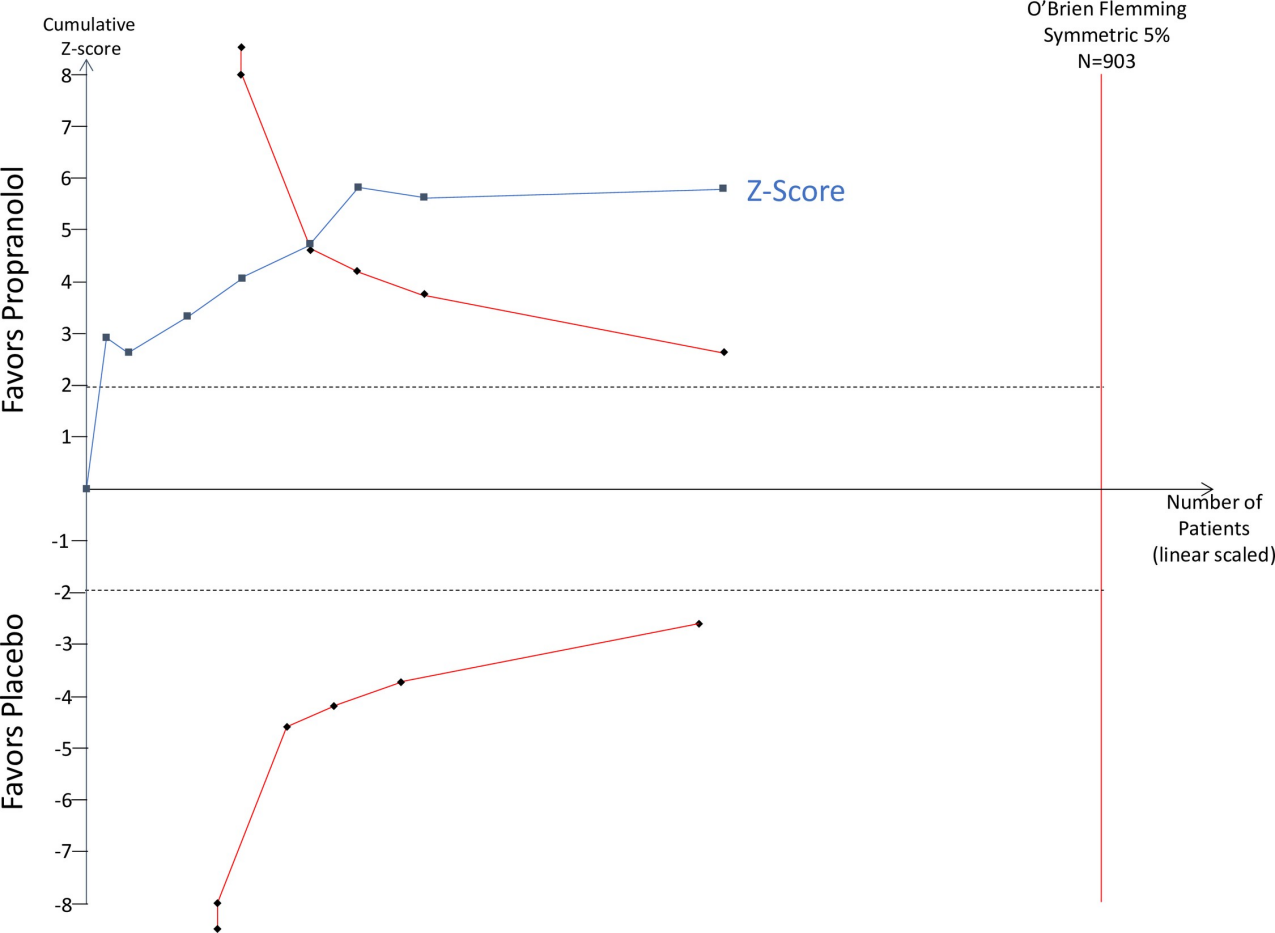
Trial Sequential Analysis (Propranolol v. Placebo).

**Fig 4:**
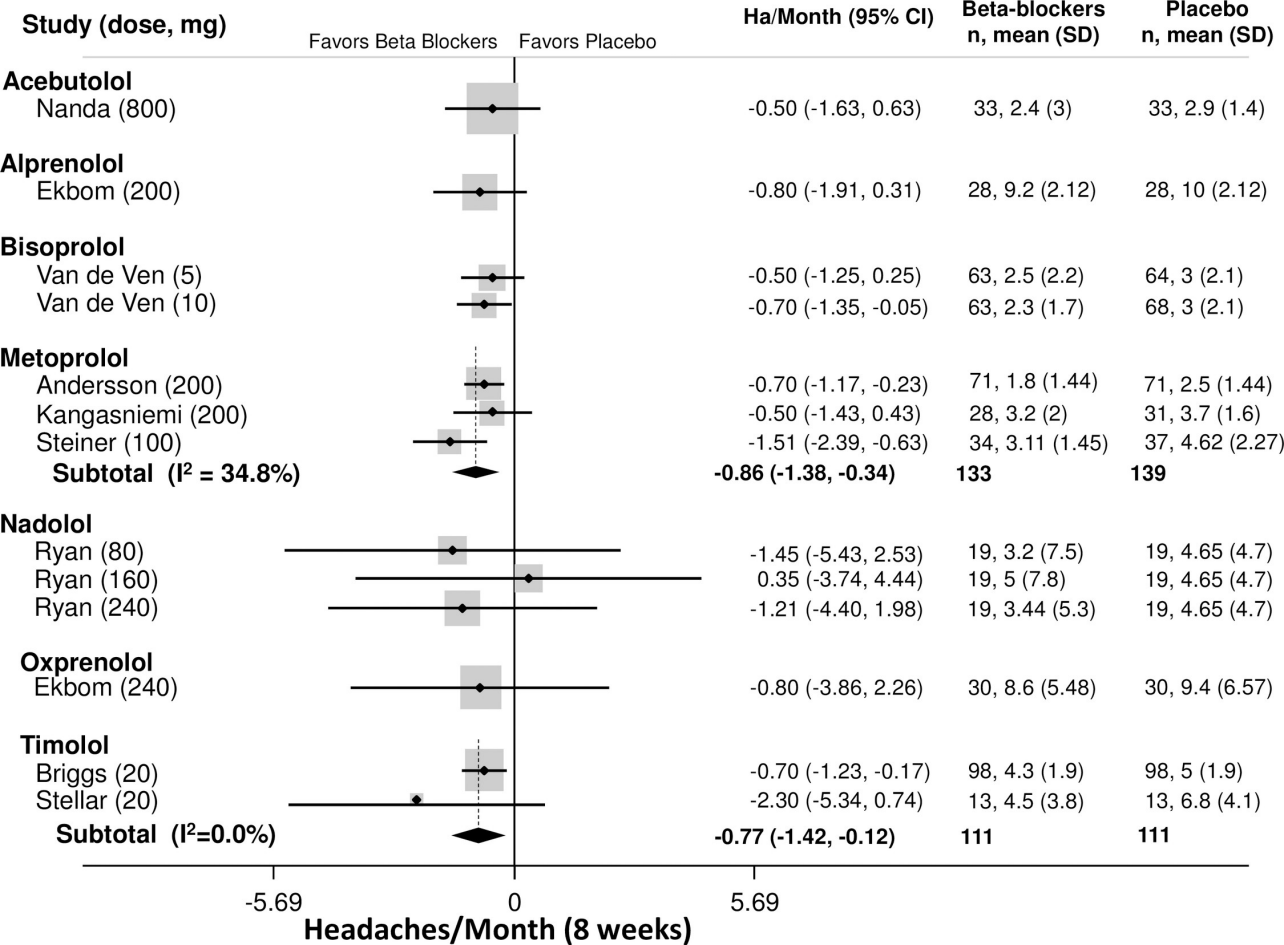
Headaches per month, other Beta Blockers v Placebo.

The primary outcome was headaches per month. Outcomes from placebo-controlled trials for all beta-blockers and time-points are provided in Tables 2–3. Outcomes at 8 weeks was the most commonly reported time point. Among patients with episodic migraines (Table 2), the average number of headaches at baseline was 4.9 headaches/month (95% CI: 4.4–5.4). The best studied beta-blocker was propranolol, which was more effective than placebo at 8 and 12 weeks (8 weeks: -1.5 ha/month, 95% CI: -2.3 to -0.65); 12 weeks: -1.2 ha/month, 95% CI: -1.8 to -0.60, Fig 2). Propranolol outcomes at 8 and 12 weeks were both graded as high-quality evidence. TSA analysis of propranolol vs. placebo for headache frequency demonstrated that it is unlikely that these results are due to a Type 1 error (Fig 3). Other beta-blockers that were more effective than placebo at 8 weeks (Fig 4) included bisoprolol (-0.70 ha/month, 95% CI: -1.4 to -0.05, low quality), metoprolol (-0.86 ha/month, 95% CI: -1.4 to -0.34, moderate quality) and timolol (-0.77 ha/month, 95% CI: -1.4 to -0.12, moderate quality). The remaining beta-blockers, in single trials did not significantly reduce headache frequency (Fig 3). There was a similar pattern at twelve weeks.

**Table 2:** Placebo controlled primary outcome (headache frequency per month).

Episodic Migraines			
Time point	Weighted Mean Difference (95% CI)	Heterogeneity	Quality of Evidence (GRADE)
**Acebutolol** (HA frequency at baseline: 4.8 headaches/month (95% CI: -0.64 to 8.9))			
Baseline Frequency	0.00 (-0.14 to 0.14)	—	Low
4 weeks	-0.20 (-0.38 to -0.02)	—	
8 weeks	-0.50 (-1.6 to 0.63)	—	
12 weeks	-0.6 (-1.7 to 0.53)	—	
**Alprenolol**			
8 weeks	-0.80 (-1.9 to 0.31)	—	Low
**Atenolol**			
12 weeks	-1.7 (-3.0 to -0.32)	—	Low
**Bisoprolol** (HA frequency at baseline: 5.5 headaches/month (95% CI: 2.7 to 8.3))			
Baseline			Low
5 mg	0.40 (-0.19 to 0.99)	—	
10 mg	0.20 (-0.40 to 0.80)	—	
4 weeks			
5 mg	-0.40 (-1.1 to 0.29)	—	
10 mg	-0.40 (-1.1 to 0.34)	—	
8 weeks			
5 mg	-0.50 (-1.2 to 0.25)	—	
10 mg	-0.70 (-1.4 to -0.05)	—	
12 weeks			
5 mg	-0.90 (-1.53 to -0.27)	—	
10 mg	-0.90 (-1.6 to -0.24)	—	
**Metoprolol** (HA frequency at baseline: 3.9 headaches/month (3.1 to 4.7))			
Baseline	-0.04 (-0.49 to 0.41)	Q = 3.68, df = 3, I^2^ = 18.5%	Low
4 weeks	-0.91 (-2.6 to 0.82)	—	—
8 weeks	-0.86(-1.4 to -0.34)	Q = 3.07, df = 2, I^2^ = 34.8%	Moderate
12 weeks	-0.90 (-2.2 to 0.41)	—	Low
**Nadolol** (HA frequency at baseline: 6.7 headaches/month (3.4 to 9.9)			
Baseline	0.22 (-1.8 to 2.3)	—	Low
4 weeks	1.1 (-0.98 to 3.2)	—	
8 weeks	-0.86 (-2.9 to 1.3)	—	
12 weeks	-0.96 (-3.1 to 1.2)	—	
**Oxprenolol**			
8 weeks	-0.80 (-3.9 to 2.3)	—	Low
**Propranolol** (HA frequency at baseline: 4.8 headaches/month (4.3 to 5.3))—			
Baseline	-0.04 (-0.28 to 0.20)	Q = 0.83, df = 10, I^2^ = 0.0%	High
4 weeks	-1.1 (-1.8 to -0.43)	Q = 0.0, df = 1, I2 = 0.0%	Moderate
8 weeks	-1.5 (-2.3 to -0.65)	Q = 11.37, df = 7, I2 = 38.4%	High
12 weeks	-1.2 (-1.8 to-0.60)	Q = 35.29, df = 8, I2 = 77.3%	High
16 weeks	-1.2 (-2.4 to -0.01)	—	Low
20 weeks	-0.9 (-1.8 to -0.02)	—	Low
24 weeks	-0.9 (-1.5 to -0.32)	—	Low
40 weeks	-0.3 (-0.9 to 0.34)	—	Low
64 weeks	-0.3 (-0.98 to 0.38)	—	Low
**Timolol** (HA frequency at baseline: 4.8 headaches/month (95% CI: -0.64 to 8.9))			
Baseline	(-0.45 to 0.45)	Q = 0.0, df = 2, I2 = 0.0%	Moderate
8 weeks	-0.77 (-1.4 to -0.12)	Q = 1.03, df = 1, I^2^ = 3.2%	
12 weeks	-1.53 (-2.5 to -0.78)	Q = 0.16, df = 1, I^2^ = 0.0%	
**Migraine-Chronic**			
**Propranolol**			
8 weeks	-2.1 (-5.5 to 1.3)	—	Low
**Chronic Tension-type HA**			
**Pindolol+ Amitriptyline** (HA frequency at baseline: 20.0 headaches/month (95% CI: 18.5 to 29.5))			
Baseline	1.4 (-2.2 to 5.0)	—	Low
4 weeks	-7.8 (-13.9 to -1.5)	—	
8 weeks	-11.2 (-16.7 to -5.5)	—	
**Propranolol** (HA frequency at baseline: 20.0 headaches/month (95% CI: 18.5 to 29.5))			
Baseline	(-8.1 to 8.1)	—	Low
8 weeks	-4.5 (-8.2 to -0.82)	—	

**Table 3:** Secondary outcomes of placebo controlled trials.

**Episodic Migraines**			
**50% Improvement in Headaches**			
**Beta-blocker**	**RR (95% CI)/NNT**	**Heterogeneity**	**Quality of Evidence**
Atenolol	1.8 (1.0 to 3.2)/6.3 (3.2–33.3)	—	Low
Metoprolol	1.7 (1.0 to 2.9)/5 (3.5–8.8)	Q = 8.85, df = 3, I^2^ = 66.1%	Moderate
Nadolol	5.1 (0.32 to 81.3)/3.7 (1.9–90.9)	—	Low
Propranolol	1.4 (1.1 to 1.8)/5.3 (3.4–11.4)	Q = 26.1, df = 10, I^2^ = 59.5%	High
Timolol	1.8 (1.4 to 2.3)/4.5 (3.1–7.7)	Q = 0.86, df = 2, I2 = 0.0%	Moderate
**Analgesic Medication Consumption**			
**Beta-blocker** **Time point**	**Weighted Mean Difference (95% CI)**	**Heterogeneity**	
**Metoprolol** (Baseline analgesic doses/month: 6.7, 95% CI: 3.4 to 10.0)			
Baseline	-0.17 (-1.4 to 1.1)	Q = 0.5, df = 3, I^2^ = 0.0%	High
4 weeks	-2.4 (-4.9 to 0.08)	—	Low
8 weeks	-4.0 (-7.5 to -0.48)	Q = 3.49, df = 2, I^2^ = 42.8%	Moderate
**Propranolol** (Baseline analgesic doses/month 11.1, 95% CI: 4.8–17.4)			
Baseline	0.0 (-1.9 to 1.9)	Q = 0.26, df = 7, I^2^ = 0.00	High
4 weeks	-6.0 (-11.8 to -0.12)	—	Low
8 weeks	-2.9 (-25.9 to 20.2)	—	Low
12 weeks	-2.1 (-3.2 to -0.95)	Q = 33.82, df = 5, I^2^ = 85.2%	High
**Headache Index**			
**Beta-blocker** **Time point**	**Standardized Mean Difference (95% CI)**	**Heterogeneity**	
**Alprenolol**			
8 weeks	0.05 (-0.47 to 0.58)	—	Low
**Atenolol**			
8 weeks	-0.62 (-1.2 to -0.004)	—	Low
12 weeks	-0.65 (-1.3 to -0.01)	—	
**Metoprolol**			
Baseline	0.12 (-0.22 to 0.47)	Q = 0.07, df = 1, I^2^ = 0.0%	Moderate
8 weeks	-0.42 (-0.77 to -0.07)	Q = 0.98, df = 1, I^2^ = 0.0%	
**Nadolol**			
Baseline	0.12 (-0.24 to 0.48)	—	Low
4 weeks	0.16 (-0.20 to 0.52)	—	
8 weeks	-0.14 (-0.50 to 0.23)	—	
12 weeks	-0.27 (-0.64 to 0.10)	—	
**Oxprenolol**			
4 weeks	-1.7 (-2.3 to -1.1)	—	Low
8 weeks	-0.42 (-0.09 to 0.93)	—	
**Pindolol**			
Baseline	0.14 (-0.52 to 0.80)	—	Low
4 weeks	0.27 (-0.40 to 0.93)	—	
**Propranolol**			
0 weeks	-0.03 (-0.23 to 0.16)	Q = 6.24, df = 6, I^2^ = 19.9%	High
4 weeks	-0.66 (-1.3 to -0.01)	—	Low
8 weeks	-0.48 (-0.75 to -0.22)	Q = 2.54, df = 4, I^2^ = 0.0%	High
12 weeks	-0.41 (-0.65 to -0.17)	Q = 2.56, df = 3, I^2^ = 0.0%	High
**Timolol**			
Baseline	0.0 (-0.31 to 0.31)	—	Low
12 weeks	-0.53 (-0.84 to -0.21)	—	
**Headache Severity**			
**Metoprolol**			
Baseline	-0.06 (-0.33 to 0.21)	Q = 1.65, df = 2, I^2^ = 0.0%	High
8 weeks	-0.53 (-0.71 to -0.14)	Q = 0.15, df = 1, I^2^ = 0.0%	Moderate
**Propranolol**			
Baseline	(-0.21 to 0.21)	Q = 0.00, df = 2, I^2^ = 0.0%	High
8 weeks	-0.51 (-0.76 to -0.26)	Q = 1.73, df = 4, I^2^ = 0.0%	
12 weeks	0.18 (-0.30 to 0.01)	Q = 3.70, df = 2, I^2^ = 46.0%	
**Timolol**			
Baseline	0.00 (-0.31 to 0.31)	—	Low
12 weeks	-0.35 (-0.66 to -0.04)	—	
**Headache Duration (hours)**			
**Beta-blocker** **Time point**	**Weighted Mean Difference (95% CI)**	**Heterogeneity**	
**Bisoprolol (baseline duration 20.6 hours, 95% CI: 0 to 46.7)**			
Baseline	-3.9 (-8.6 to 0.77)	—	Low
12 weeks	-1.9 (-6.5 to 2.5)	—	
**Metoprolol (baseline duration: 11.6 hours, 95% CI: 2.6 to 20.5)**			
Baseline	0.06 (-1.6 to 1.7)	—	Low
4 weeks	-2.6 (-4.2 to -0.88)	—	
8 weeks	-2.0 (-3.7 to -0.26)	—	
**Propranolol (baseline duration: 28.9 hours, 95% CI: 0 to 72.3)**			
Baseline	0.22 (-2.4 to 2.8)	Q = 0.99, df = 2, I^2^ = 0.0%	High
8 weeks	-6.1 (-16.2 to -0.39)	Q = 5.67, df = 2, I^2^ = 64.7%	
12 weeks	-1.6 (-3.0 to -0.11)	Q = 2.50, df = 4, I^2^ = 0.0%	
**Pindolol (baseline duration: 6.6 hours, 95% CI: 0 to 23.7)**			
Baseline	-0.93 (-9,7 to 7.8)	Q = 0.01, df = 1, I^2^ = 0.0%	Moderate
4 weeks	-0.18 (-8.8 to 8.4)	Q = 0.01, df = 1, I^2^ = 0.0%	
**Timolol (baseline duration 9.4 hours, 95% CI: 0 to 19.8)**			
Baseline	(-1.3 to 1.3)	—	Low
8 weeks	-0.70 (-2.2 to 0.80)	—	
12 weeks	-0.54 (-2.7 to 1.6)	—	
**Tension-type Headache**			
**50% improvement in Headache**			
**Beta-blocker**	**RR (95% CI)**	**Heterogeneity**	
Pindolol + Amitriptyline	3.8 (1.5 to 9.3)	—	Low
**Headache Index**			
**Propranolol**			
4 weeks	-0.52 (-1.0 to -0.003)	—	Low
**Severity**			
**Pindolol + Amitriptyline**			
Baseline	-0.29 (-0.34 to 0.91)	—	Low
8 weeks	-0.68 (-1.3 to -0.04)	—	

Among secondary outcomes, the majority of trials studied subjects with episodic migraine headaches (Table 3). Propranolol was the most commonly studied beta-blocker. Propranolol was more likely to reduce headaches by 50% than placebo at 12 weeks (RR: 1.4, 95% CI: 1.1–1.8, NNT: 4.5, 95% CI: 2.8–12.9). Other effective beta-blockers included atenolol (RR: 1.8, 95% CI: 1.0–3.2, NNT: 6.3, 95% CI: 3.2–332.4), metoprolol (RR: 1.9, 95% CI: 1.3–2.8, NNT: 4.7, 95% CI:3.0–10.4) and timolol (RR: 1.8, 95% CI: 1.4–2.3, NNT: 4.2, 95% CI: 2.7–8.8,). At 8 weeks, metoprolol reduced analgesic medication use (-4.0 doses/month, 95% CI: -7.5 to -0.48) as did propranolol at 12 weeks (-2.1 doses/month, 95% CI: -3.2 to -0.95). The headache index was modestly reduced by a number of different beta-blockers including atenolol (SMD: -0.62, 95% CI: -1.2 to -0.004), metoprolol (SMD: -0.42, 95% CI: -0.77 to -0.07), propranolol (SMD: -0.48, 95% CI: -0.75 to -0.22) and timolol (SMD: -0.53, 95% CI: -0.84 to -0.21). At 8 weeks, headache severity was modestly reduced by both metoprolol (SMD: -0.53, 95% CI: -0.71 to -0.14) and propranolol (SMD: -0.51, 95% CI: -0.76 to -0.26). Headache duration was reduced by metoprolol (-2.0 hours, 95% CI: -3.7 to -0.26) and propranolol (-6.1 hours, 95% CI: -16.2 to -0.39).

#### Network meta-analysis

**Fig 5:**
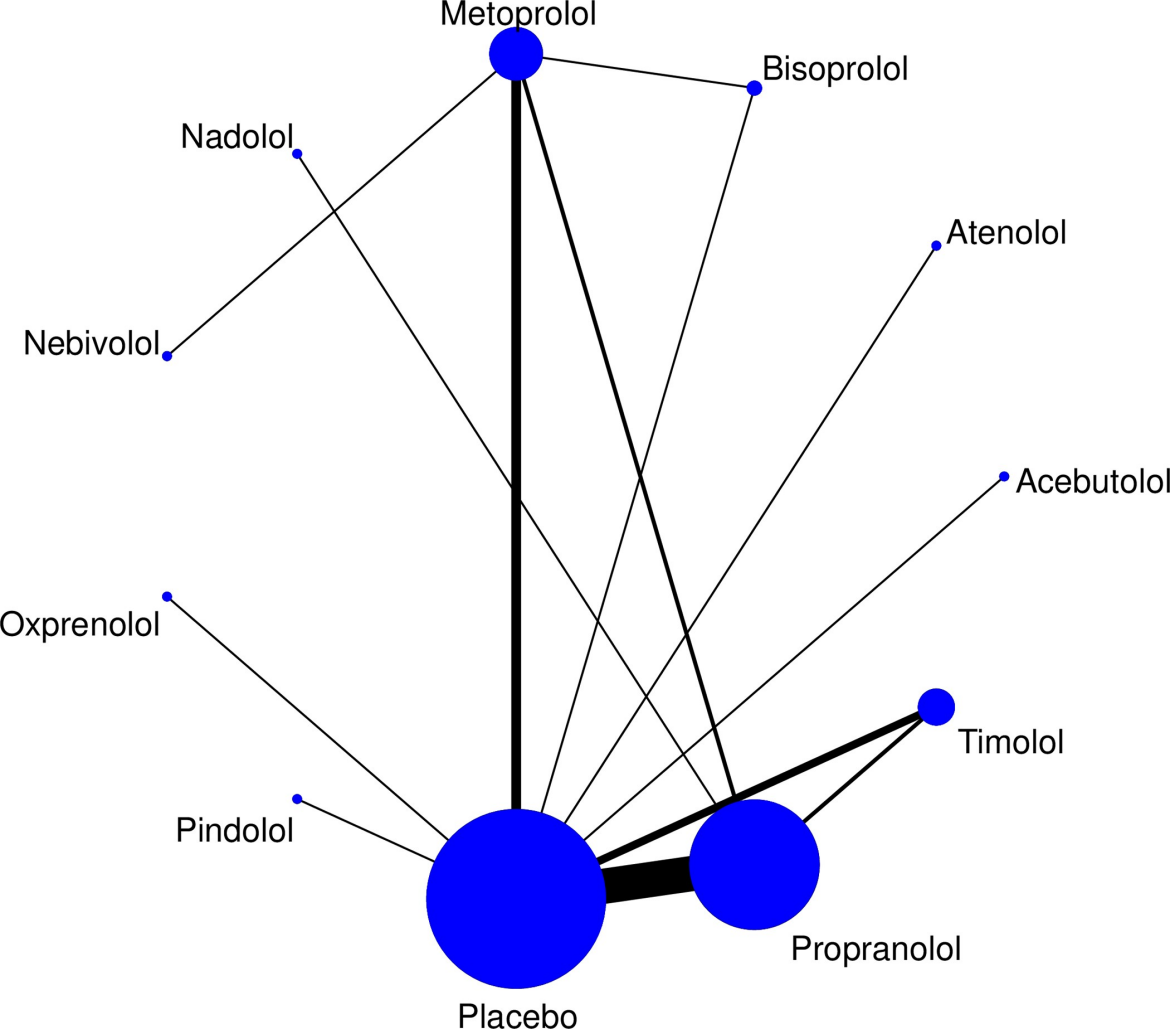
Network meta-analysis map.

**Fig 6:**
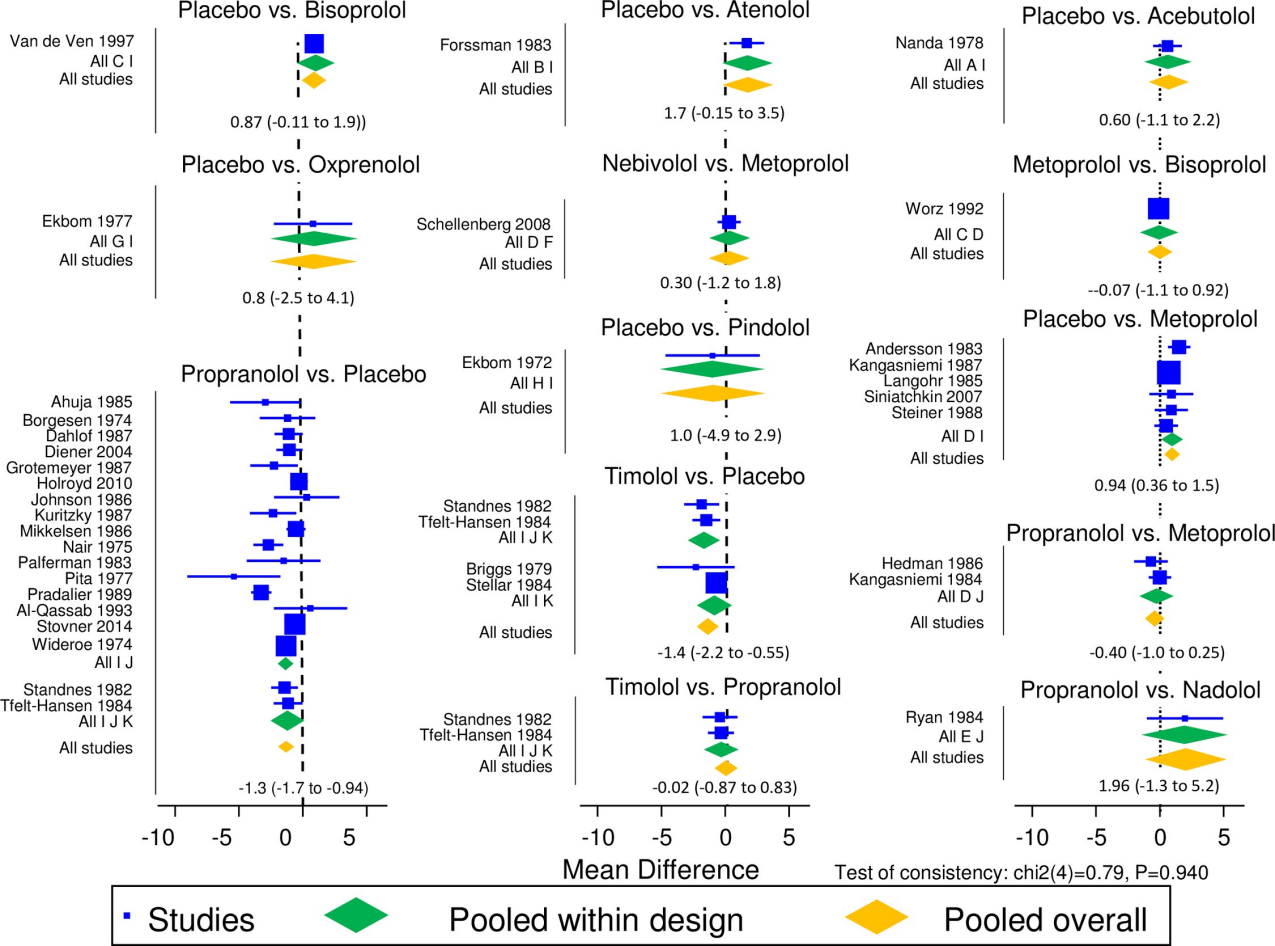
Results of network meta-analysis comparisons.

For the primary outcome, headache frequency, the network meta-analysis found no difference at 8 weeks (p = 0.27) in effectiveness in comparisons between propranolol (n = 9) compared to bisoprolol (n = 2), metoprolol (n = 3) and nadolol (n = 3). Similarly, at 12 weeks, there was no difference (p = 0.84) n effectiveness in comparisons between propranolol (n = 9) compared to bisoprolol (n = 2), nadolol (n = 3) and timolol (n = 2). The 8- and 12-week analysis confirmed this lack of difference between all beta-blockers (Fig 5), including those with single trials (Fig 6)

#### Comparative effectiveness trials

There were 83 randomized trials that included at least one comparison to a non-placebo treatment. Propranolol was the most commonly compared beta-blocker (n = 72, 87%). Propranolol was compared to pharmacologic interventions in 50 trials. Eleven comparisons that were from single trials (5-hydroxytryptophan, aspirin, atenolol, candesartan, clonidine, cyclandelate, mefenamic acid, naproxen, nifedipine, nimodipine, nortriptyline, pindolol, riboflavin). Comparisons with more than one study included: amitriptyline (n = 2), femoxetine (n = 2), flunarizine (n = 11), metoprolol (n = 4), nadolol (n = 3), nimodipine (n = 2), timolol (n = 2) tofenamic acid (n = 2), topiramate (n = 3) and valproate (n = 3). Several combinations were tested including propranolol + amitriptyline vs. amitriptyline (n = 1), and propranolol + flunarizine was compared to flunarizine. Propranolol combined with topiramate was compared with topiramate alone. Nonpharmacologic interventions were compared to propranolol in three trials (acupuncture, behavioral management, biofeedback). Metoprolol was assessed in 14 trials, 8 were comparisons with pharmacologic interventions (aspirin, bisoprolol, clomipramine, clonidine, flunarizine, nebivolol, pizotifen) and one with nonpharmacologic (acupuncture). Metoprolol was compared to a combination of metoprolol and fluoxetine in 2 trials and the combination of metoprolol and flunarizine was compared to flunarizine alone in 2 trials.

The primary outcome comparison (headache frequency) is provided in Table 4, and secondary outcomes are in Table 5. The majority of comparisons were single trials, making definitive conclusions difficult. Metoprolol, but not propranolol, was more effective than aspirin. Metoprolol was more effective than clomipramine, though comparable to acupuncture, bisoprolol, clonidine, flunarizine, nebivolol and pizotifen. Adding fluoxetine to metoprolol or flunarizine to either propranolol or metoprolol did not improve headache frequency. All comparisons were graded as low-quality. Propranolol was more effective than femoxetine, mefenamic acid, naproxen, nifedipine and very low-dose (40mg) nortriptyline, but comparable to 5-hydroxytryptophan, acupuncture, atenolol, behavioral management, biofeedback, candesartan, clonidine, cyclandelate, flunarizine, metoprolol, nadolol, naproxen, nimodipine, riboflavin, timolol, tolfenamic acid, topiramate and valproic acid. All comparisons were single trials and were rated as low-quality evidence with the exception of the comparisons to flunarizine and metoprolol at 8 weeks that were graded as moderate or high-quality. The network meta-analysis confirmed these findings, but suggested that metoprolol was also superior to naproxen (SMD: -1.2, 95% CI: -1.6 to -0.78).

**Table 4:** Primary outcome (headache frequency) of comparative effectiveness trials.

Comparison, Time point		Weighted Mean Difference (95% CI)	Heterogeneity	Quality of Evidence
**Bisoprolol (5mg)**	**Bisoprolol (10mg)**			
	Baseline	0.20 (-0.43 to 0.83)	—	Low
	4 Weeks	0.0 (-0.74 to 0.74)	—	
	8 weeks	0.20 (-0.47 to -0.87)	—	
	12 weeks	0.0 (-0.67 to -0.67)	—	
**Metoprolol**	**Acupuncture**			
	17 weeks	-0.7 (-2.7 to 1.3)	—	Low
	**Aspirin**			
	8 weeks	-1.6 (-2.8 to -0.46)	—	Low
	**Bisoprolol**			
	Baseline	0.0 (-0.31 to 0.31)	—	Low
	12 weeks	-0.09 (-0.62 to 0.44)	—	
	**Clomipramine**			
	4 weeks	-2.0 (-3.9 to -0.16)	—	Low
	**Clonidine**			
	Baseline	0.00 (-0.51 to 0.51)	—	Low
	8 weeks	-1.40 (-3.3 to 0.44)	—	
	**Flunarizine**			
	Baseline	-0.60 (-1.4 to 0.19)	—	Low
	4 weeks	-0.3 (-1.1 to 0.53)	—	
	8 weeks	-0.9 (-1.7 to -0.10)	—	
	12 weeks	-0.5 (-1.3 to 0.33)	—	
	16 weeks	-0.1 (-0.93 to 0.73)	—	
	20 weeks	-0.36 (-1.5 to 0.75)	—	
	**Metoprolol + Fluoxetine**			
	Baseline	-0.03 (-0.40 to 0.33)	—	Low
	8 weeks	0.30 (0.19 to 0.40)	—	
	**Nebivolol**			
	Baseline	0.10 (-0.62 to 0.82)	—	Low
	16 weeks	-0.30 (-1.2 to 0.60)	—	
	**Pizotifen**			
	Baseline	0.00 (-0.47 to 0.47)	—	Low
	8 weeks	0.90 (-0.01 to 1.8)	—	
**Metoprolol + Flunarizine**	**Flunarizine**			
	12 weeks	-0.9 (-1.6 to -0.22)	—	Low
	24 weeks	-0.8 (-1.6 to -0.04)	—	
	36 weeks	-0.6 (-2.4 to 1.2)	—	
	48 weeks	-0.3 (-1.2 to 0.57)	—	
**Nadolol (80mg)**	**Nadolol (160mg)**			
	Baseline	0.57 (-2.1 to 3.3)	—	Low
	4 weeks	-0.25 (-2.9 to 2.4)	—	
	8 weeks	0.11 (-2.6 to 2.8)	—	
	12 weeks	-0.34 (-3.1 to 2.4)	—	
**Pindolol**	**Pindolol (15mg)**			
	Baseline	4.0 (-0.6 to 8.1)	—	Low
	4 weeks	4.0 (-0.05 to 8.1)	—	
**Propranolol**	**5-Hydroxy-tryptophan**			
	Baseline	-2.3 (-5.9 to 1.3)	—	Low
	16 weeks	-2.9 (-6.6 to 0.81)	—	
	**Acupuncture**			
	Baseline	-0.10 (-0.59 to 0.40)	—	Low
	12 weeks	0.30 (-0.25 to 0.85)	—	
	**Behavioral Management**			
	Baseline	-0.40 (-1.1 to 0.34)	—	Low
	40 weeks	-0.30 (-0.94 to 0.34)	—	
	64 weeks	-0.20 (-0.89 to 0.49)	—	
	**Propranolol + Behavioral Management**			
	Baseline	-0.50 (-1.2 to 0.18)	—	Low
	40 weeks	0.70 (-.16 to 1.2)	—	
	64 weeks	0.80 (0.19 to 1.4)	—	
	**Biofeedback**			
	24 weeks	0.53 (0.08 to 0.97)	—	Low
	**Candesartan**			
	12 weeks	-0.04 (-0.59 to 0.51)	—	Low
	**Cyclandelate (HA days/mo)**			
	Baseline	-0.16 (-0.66 to 0.35)	—	Low
	4 weeks	-0.32 (-0.83 to 0.18)	—	
	8 weeks	0.13 (-0.37 to 0.63)	—	
	**Femoxetine**			
	Baseline	0.0 (-2.2 to 2.2)	—	Low
	8 weeks	-0.70 (-1.5 to 0.13)	—	
	12 weeks	-1.5 (-3.6 to 0.55)	—	
	**Flunarizine**			
	Baseline	-0.005 (-0.11 to 0.10)	Q = 0.83, df = 5, I^2^ = 0.0%	High
	4 weeks	0.40 (-0.34 to 1.1)	Q = 3.1, df = 3, I^2^ = 3.7%	High
	8 weeks	0.42 (-0.55 to 1.4)	Q = 1.48, df = 1, I^2^ = 32.6%	Moderate
	12 weeks	0.68 (-0.06 to 1.4)	Q = 0.55, df = 2, I^2^ = 0.0%	High
	16 weeks	-0.04 (-0.19 to 0.12)	Q = 5.44, df = 6, I^2^ = 7.0%	High
	**Mefenamic Acid**			
	12 weeks	-2.1 (-5.3 to 1.3)	—	Low
	**Metoprolol**			
	Baseline	0.00 (-0.38 to 0.38)	—	Low
	4 weeks	-0.70 (-2.0 to 0.62)	—	
	8 weeks	0.00 (-0.54 to 0.56)	Q = 0.0, df = 1, I^2^ = 0.0%	
	**Nadolol**			
	Baseline	0.27 (-2.6 to 3.2)	—	Low
	4 weeks	1.6 (-0.28 to 3.4)	—	
	8 weeks	1.7 (-0.23 to 3.6)	—	
	12 weeks	1.8 (-0.16 to 3.7)	—	
	24 weeks	-4.8 (-8.9 to -0.77)	—	
	**Naproxen**			
	12 weeks	-1.4 (-1.9 to -0.95)	—	Low
	**Nifedipine**			
	Baseline	2.5 (1.0 to 3.9)	—	Low
	12 weeks	-0.40 (-2.6 to 1.8)	—	
	24 weeks	0.70 (-1.5 to 2.9)	—	
	**Nimodipine**			
	Baseline	0.20 (-0.86 to 1.2)	—	Low
	4 weeks	1.6 (-0.24 to 3.4)	—	
	8 weeks	-1.5 (-4.1 to 1.1)	—	
	12 weeks	-1.5 (-2.9 to -0.04)	—	
	16 weeks	-0.30 (-1.7 to 1.1)	—	
	24 weeks	-0.01 (-1.3 to 1.3)	—	
	**Propranolol + Flunarizine**			
	Baseline	-0.14 (-1.8 to 1.5)	—	Low
	16 weeks	0.18 (-1.4 to 1.8)	—	
	**Riboflavin**			
	Baseline	0.00 (-0.39 to 0.39)	—	Low
	4 weeks	-0.60 (-0.92 to -0.28)	—	
	8 weeks	-0.10 (-0.42 to 0.22)	—	
	12 weeks	0.0 (-0.27 to 0.27)	—	
	24 weeks	-0.10 (-0.37 to 0.17)	—	
	**Timolol**			
	Baseline	0.00 (-0.84 to 0.84)	Q = 0.00, df = 1 I^2^ = 0.0%	Moderate
	12 weeks	0.37 (-0.45 to 1.2)	Q = 0.01, df = 1, I^2^ = 0.0%	
	**Tolfenamic Acid**			
	12 weeks	0.01 (-0.68 to 0.70)	—	Low
	**Topiramate**			
	Baseline	-0.02 (-0.35 to 0.31)	Q = 1.47, df = 2 I^2^ = 0.0%	Moderate
	4 weeks	-0.19 (-0.76 to 0.39)	Q = 0.18, df = 1 I^2^ = 0.0%	
	8 weeks	0.30 (-0.30 to 0.91)	—	Low
	12 weeks	0.10 (-0.98 to 1.2)	—	
	16 weeks	0.30 (-0.94 to 1.5)	—	
	20 weeks	0.40 (-0.60 to 1.4)	—	
	24 weeks	-0.75 (-1.6 to 0.13)	Q = 4.38, df = 1 I^2^ = 77.2%	
	**Valproic Acid**			
	Baseline	-0.20 (-1.1 to 0.71)	—	Low
	8 weeks	-0.16 (-1.7 to 0.75)	—	
**Propranolol + Flunarizine**	**Topiramate**			
	Baseline	-0.06 (-0.82 to 0.70)	—	Low
	12 weeks	0.70 (0.29 to 1.1)	—	
**Propranolol + Topiramate**	**Topiramate**			
	24 weeks	-0.01 (-2.4 to 2.1)	—	Low

**Table 5:** Secondary outcomes of comparative effectiveness trials.

Drug1	Comparison, Time Point	Weighted Mean Difference (95% CI)	Heterogeneity	Quality of Evidence
**Headache Days Weighted Mean Difference (95% CI)**				
**Metoprolol**	**Clonidine**			
	Baseline	0.0 (-3.7 to 3.7)	—	Low
	8 Weeks	-1.8 (-5.7 to 2.14)	—	
	**Metoprolol + Fluoxetine**			
	8 weeks	0.83 (0.63 to 1.0)	—	Low
**Nadolol (80mg)**	**Nadolol (160mg)**			
	Baseline	2.3 (-3.4 to 7.9)	—	Low
	4 weeks	-0.22 (-5.9 to 5.4)	—	
	8 weeks	-0.05 (-5.9 to 5.8)	—	
	12 weeks	2.2 (-7.9 to 3.6)	—	
**Pindolol (7.5mg)**	**Pindolol (15mg)**			
	Baseline	0.05 (-0.94 to 1.0)	—	Low
	4 weeks	0.28 (-0.72 to 1.3)	—	
**Propranolol**	**Acupuncture**			
	Baseline	0.0 (-1.0 to 1.0)	—	Low
	12 weeks	0.70 (-0.43 to 1.8)	—	
	**Atenolol**			
	8 weeks	0.06 (-1.8 to 1.9)	—	Low
	**Behavioral Management**			
	Baseline	0.50 (-0.88 to 1.9)	—	Low
	40 weeks	-0.10 (-1.3 to 1.1)	—	
	64 weeks	0.10 (-1.1 to 1.3)	—	
	**Candesartan**			
	12 weeks	0.45 (-1.2 to 2.1)	—	Low
	**Clonidine**			
	16 weeks	0.10 (-2.1 to 2.3)	—	Low
	**Cyclandelate**			
	Baseline	-0.60 (-2.5 to 1.3)	—	Low
	4 weeks	-1.1 (-2.8 to 0.64)	—	
	8 weeks	0.40 (-1.2 to 2.0)	—	
	**Femoxetine**			
	8 weeks	-1.6 (-4.5 to 1.3)	—	Low
	**Flunarizine**			
	Baseline	-0.80 (-2.2 to 0.59)	—	Low
	4 weeks	1.4 (0.02 to 2.8)	—	
	8 weeks	0.50 (-0.91 to 1.9)	—	
	12 weeks	-0.5 (-1.9 to 0.96)	—	
	16 weeks	0.61 (-0.91 to 2.1)	—	
	**Metoprolol**			
	Baseline	-0.31 (-1.3 to 0.68)	—	Low
	4 weeks	-0.60 (-4.3 to 3.1)	—	Low
	8 weeks	-0.17 (-0.96 to 0.51)	Q = 0.73 df = 1, I^2^ = 0.0%	Moderate
	16 weeks	0.06 (-1.9 to 2.02)	—	Low
	24 weeks	-2.2 (-4.2 to -0.24)	—	Low
	**Naproxen**			
	12 weeks	-2.8 (-3.6 to -1.9)	—	Low
	**Nifedipine**			
	Baseline	-0.80 (-3.3 to 1.7)	—	Low
	4 weeks	-3.6 (-7.0 to -0.16)	—	
	16 weeks	-2.2 (-4.0 to -0.32)	—	
	28 weeks	-0.80 (-2.6 to 1.0)	—	
	**Propranolol (80mg v 160mg)**			
	Baseline	0.0 (-1.7 to 1.7)	—	Low
	12 weeks	-2.7 (04.9 to -0.5)	—	
	**Tolfenamic Acid**			
	Baseline	0.0 (-1.6 to 1.6)	—	Low
	12 weeks	-0.22 (-2.1 to 1.6)	—	
	**Topiramate**			
	Baseline	0.12 (-0.3 to 0.55)	—	Low
	24 weeks	-0.25 (01.13 to 0.63)	—	
**Headache Index, Standardized Mean Difference (95% CI)**				
**Metoprolol**	**Clonidine**			
	Baseline	0.0 (-0.49 to 0.49)	—	Low
	8 Weeks	-0.24 (-0.76 to 0.29)	—	
**Pindolol (7.5mg)**	**Pindolol (15 mg)**			
	Baseline	0.05 (-0.94 to 1.04)	—	Low
	4 weeks	0.27 (-0.72 to 1.3)	—	
**Propranolol**	**Amitriptyline**			
	Baseline	0.05 (-0.42 to 0.52)	—	Low
	6 weeks	-0.42 (-0.89 to 0.06)	—	
	**Amitriptyline + Biofeedback**			
	Baseline	0.08 (-0.37 to 0.53)	—	Low
	6 weeks	-0.24 (-0.69 to 0.21)	—	
	**Amitriptyline + Propranolol**			
	Baseline	0.01 (-0.44 to 0.46)	—	Low
	6 weeks	0.06 (-0.39 to 0.51)	—	
	**Amitriptyline + Propranolol + Biofeedback**			
	Baseline	-0.04 (-0.52 to 0.44)	—	Low
	6 weeks	0.25 (-0.23 to 0.74)	—	
	**Biofeedback**			
	Baseline	0.17 (-0.31 to 0.64)	—	Low
	6 weeks	-0.35 (-0.83 to 0.13)	—	
	**Biofeedback + Propranolol**			
	Baseline	-0.03 (-0.50 to 0.44)	—	Low
	6 weeks	0.39 (-0.09 to 0.86)	—	
	**Aspirin**			
	Baseline	0.00 (-0.65 to 0.65)	—	Low
	6 weeks	0.25 (-0.56 to 1.05)	—	
	**Atenolol**			
	6 weeks	0.01 (-0.59 to 0.62)	—	Low
	**Femoxetine**			
	Baseline	0.00 (-0.57 to 0.57)	—	Low
	8 weeks	-0.24 (-0.70 to 0.21)	—	
	12 weeks	-0.35 (-0.92 to 0.22)	—	
	**Flunarizine**			
	Baseline	0.12 (-0.17 to 0.42)	Q = 1.59, df = 2, I^2^ = 0.0%	Moderate
	4 weeks	-0.18 (-0.82 to 0.46)	Q = 2.45, df = 2, I^2^ = 59.3%	
	8 weeks	-0.13 (-0.75 to 0.48)	Q = 2.27, df = 1, I^2^ = 55.9%	
	12 weeks	0.16 (-1.1 to 1.4)	Q = 8.7, df = 1, I^2^ = 88.5%	
	16 weeks	-0.08 (-0.48 to 0.32)	Q = 3.13, df = 2, I^2^ = 49.3%	
	**Flunarizine + Propranolol**			
	Baseline	0.42 (-0.30 to 1.1)	—	Low
	4 weeks	0.29 (-0.43 to 1.0)	—	
	8 weeks	0.17 (-0.54 to 0.89)	—	
	12 weeks	0.47 (-0.26 to 1.2)	—	
	16 weeks	0.67 (-0.07 to 1.4)	—	
	**Metoprolol**			
	Baseline	0.00 (-0.29 to 0.29)	Q = 0.00, df = 1, I^2^ = 0.0%	Moderate
	8 weeks	0.06 (-0.24 to 0.35)	Q = 0.23, df = 1, I^2^ = 0.0%	
	**Nadolol**			
	Baseline	0.41 (-0.09 to 0.90)	—	Low
	4 weeks	0.34 (-0.16 to 0.83)	—	
	8 weeks	0.44 (-0.08 to 0.96)	—	
	12 weeks	0.29 (-0.22 to 0.81)	—	
	**Timolol**			
	Baseline	0.00 (-0.31 to 0.31)	—	Low
	12 weeks	0.17 (-0.14 to 0.48)	—	
	**Valproic Acid**			
	Baseline	-0.09 (-0.56 to 0.38)		
	10 weeks	-0.03 (-0.50 to 0.44)	—	Low
**Propranolol + Flunarizine**	**Topiramate**			
	Baseline	0.07 (-0.36 to 0.57)	—	Low
	12 weeks	0.99 (0.53 to 1.4)	—	
**Episodic Migraine, 50% reduction in Headaches**				
**Comparison**		**RR (95% CI)**	**Heterogeneity**	**Quality**
**Metoprolol**	**ASA**	2.4 (0.88 to 6.7)	—	Low
	**Clonidine**	1.3 (0.62 to 2.9)	—	Low
	**Flunarizine**	1.1 (0.98 to 1.3)	—	Low
	**Nebivolol**	1.1 (0.56 to 2.2)	—	Low
	**Pizotifen**	0.69 (0.36 to 1.3)	—	Low
**Metoprolol + Flunarizine**	**Flunarizine**	1.3 (1.0 to 1.6)	—	Low
**Propranolol**	**ASA**	1.3 (0.88 to 1.9)	Q = 2.44, df = 1, I^2^ = 59.1%	Moderate
	**Acupuncture**	0.88 (0.57 to 1.3)	—	Low
	**Candesartan**	0.93 (0.61 to 1.4)	—	Low
	**Clonidine**	1.6 (0.86 to 3.1)	—	Low
	**Cyclandelate**	1.0 (0.71 to 1.5)	Q = 4.39, df = 2, I^2^ = 54.4%	Moderate
	**Femoxetine**	3.5 (0.43 to 29.4)	—	Low
	**Flunarizine**	1.0 (0.89 to 1.2)	Q = 3.30, df = 5, I^2^ = 0.0%	High
	**Flunarizine + Propranolol**	0.86 (0.59 to 1.2)	—	Low
	**Metoprolol**	0.86 (0.60 to 1.2)	Q = 0.11, df = 1, I^2^ = 0.0%	Moderate
	**Nadolol**	0.66 (0.27 to 1.6)	Q = 6.20, df = 2, I^2^ = 67.8%	Moderate
	**Nifedipine**	2.2 (1.3 to 3.8)	—	Low
	**Nortriptyline**	1.5 (0.54 to 4.2)	—	Low
	**Nortriptyline + Propranolol**	1.1 (0.48 to 2.7)	—	Low
	**Propranolol (80 vs. 160mg)**	0.94 (0.72 to 1.2)	—	Low
	**Timolol**	1.1 (0.84 to 1.4)	—	Low
	**Topiramate**	1.2 (0.98 to 1.4)	Q = 0.05, df = 2 I^2^ = 0.0%	Moderate
	**Valproic Acid**	0.96 (0.77 to 1.2)	Q = 0.16, df = 2, I^2^ = 0.0%	Moderate
**Propranolol + Amitriptyline**	**Amitriptyline**	1.02 (0.92 to 1.1)	—	Low
**Propranolol + Flunarizine**	**Nimodipine + Diazepam + Oryzanol**	1.5 (1.2 to 2.0)	—	Low
**Propranolol + Nadolol**	**Behavioral Management**	0.98 (0.58 to 1.7)	—	Low
**Propranolol + Nadolol**	**Behavioral Management + Propranolol**	0.44 (0.30 to 0.66)	—	Low
**Medicine Use (doses/month)**				
**Comparison**		**Weighted Mean Difference (95% CI)**	**Heterogeneity**	**Quality of evidence**
**Metoprolol**	**Bisoprolol**			
	Baseline	0.0 (-0.31 to 0.31)	—	Low
	12 weeks	0.01 (-0.30 to 0.32)	—	
	**Clomipramine**			
	4 weeks	-0.55 (-1.2 to 0.08)	—	Low
	**Clonidine**			
	Baseline	0.0 (-0.50 to 0.50)	—	Low
	8 weeks	-0.52 (-1.0 to 0.02)	—	
**Propranolol**	**5-Hydroxytryptophan**			
	Baseline	0.48 (-0.16 to 1.1)	—	Low
	16 weeks	0.22 (-0.41 to 0.85)	—	
	**Candesartan**			
	12 weeks	-0.45 (-0.82 to -0.09)	—	Low
	**Cyclandelate**			
	Baseline	-0.49 (-1.0 to 0.02)	—	Low
	4 weeks	-0.70 (-1.2 to -0.19)	—	
	8 weeks	-0.19 (-0.69 to 0.32)	—	
	**Femoxetine**			
	Baseline	0.00 (-0.57 to -0.57)	—	Low
	12 weeks	-0.83 (-1.4 to -0.24)	—	
	**Flunarizine**			
	Baseline	0.08 (-0.10 to 0.26)	Q = 1.98, df = 2, I^2^ = 0.0%	Moderate
	4 weeks	-.44 (0.02 to 0.86)	—	Low
	8 weeks	-0.15 (-0.57 to 0.28)	—	Low
	12 weeks	-0.23 (-0.43 to -0.04)	Q = 0.06, df = 1, I^2^ = 0.0%	Moderate
	16 weeks	-0.07 (-0.41 to 0.27)	Q = 0.01, df = 1, I^2^ = 0.0%	Moderate
	**Metoprolol**			
	Baseline	0.00 (-0.29 to 0.29)	Q = 0.00, df = 1, I^2^ = 0.0%	Moderate
	8 weeks	-0.19 (-0.49 to 0.11)	Q = 1.00, df = 1, I^2^ = 0.5%	
	**Nadolo**l			
	Baseline	-0.31 (-1.1 to 0.45)	—	Low
	24 weeks	-0.73 (-1.5 to 0.05)	—	
**Health Related Quality of Life**
**Comparison**		**Standardized Mean Difference (95% CI)**	**Heterogeneity**	**Quality of evidence**
**Metoprolol**	**Nebivolol** (MOS SF36)			
	Baseline	-0.21 (-0.93 to 0.51)	—	Low
	16 Weeks	-0.46 (-1.2 to 0.27)	—	
**Propranolol**	**Acupuncture** (MOS SF36)			
	Baseline	-0.19 (-0.56 to 0.19)	—	Low
	12 weeks	-0.47 (-0.84 to -0.10)	—	
	**Behavioral Management**			
	Baseline	0.14 (-0.24 to 0.52)	—	Low
	40 weeks	0.48 (0.10 to 0.86)	—	
	64 weeks	0.23 (-0.15 to 0.60)	—	
	**Behavioral Management + Propranolol**			
	Baseline	0.07 (-0.28 to 0.43)	—	Low
	40 weeks	0.68 (0.31 to 1.0)	—	
	64 weeks	0.61 (-.24 to 0.97)	—	
	**Candesartan**			
	12 weeks	-0.18 (-0.54 to 0.19)	—	Low
	**Riboflavin** (MIDAS)			
	Baseline	-0.06 (-0.46 to 0.33)	—	Low
	4 weeks	0.00 (-0.39 to 0.39)	—	
**Propranolol + Topiramate**	**Topiramate** (MIDAS)			
	24 weeks	0.01 (-0.39 to 0.41)	—	Low
**Headache Severity**				
**Comparison**		**Standardized Mean Difference (95% CI)**	**Heterogeneity**	**Quality of evidence**
**Metoprolol**	**Aspirin**			
	8 weeks	0.33 (-0.20 to 0.86)	—	Low
	**Acupuncture**			
	17 weeks	-0.46 (-0.91 to -0.002)	—	Low
	**Bisoprolol**			
	Baseline	0.00 (-0.31 to 0.31)	—	Low
	12 weeks	0.19 (-0.13 to 0.3)	—	
	**Flunarizine**			
	Baseline	0.46 (0.14 to 0.79)	—	Low
	4 weeks	0.13 (-0.22 to 0.48)	—	
	8 weeks	0.38 (0.02 to 0.73)	—	
	12 weeks	0.13 (-0.22 to 0.48)	—	
	16 weeks	0.75 (0.39 to 1.1)	—	
	20 weeks	0.42 (0.07 to 0.77)	—	
	**Nebivolol**			
	16 weeks	0.19 (-0.53 to 0.91)	—	Low
	**Pizotifen**			
	Baseline	0.00 (-0.47 to 0.47)	—	Low
	8 weeks	-0.91 (-1.4 to -0.40)	—	
**Metoprolol + Flunarizine**	**Flunarizine**			
	12 weeks	-0.25 (-0.79 to 0.26)	—	Low
	24 weeks	-0.55 (-1.1 to -0.02)	—	
	36 weeks	-0.49 (-1.0 to 0.047)	—	
	48 weeks	-0.54 (-0.72 to -0.19)	—	
**Propranolol**	**Acupuncture**			
	Baseline	0.26 (-0.13 to 0.65	—	Low
	12 weeks	0.63 (0.23 to 1.03)	—	
	**5-hydroxytryptophan**			
	Baseline	0.18 (-0.45 to 0.81)	—	Low
	16 weeks	0.00 (-0.63 to 0.63)	—	
	**Biofeedback**			
	Baseline	-0.07 (-0.36 to 0.21)	—	Low
	24 weeks	0.13 (-0.15 to 0.42)	—	
	**Cyclandelate**			
	Baseline	-0.07 (-0.58 to 0.43)	—	Low
	4 weeks	-0.17 (-0.68 to 0.33)	—	
	8 weeks	-0.01 (-0.51 to 0.48)	—	
	**Flunarizine**			
	Baseline	0.00 (-0.32 to 0.32)	Q = 0.0, df = 1, I^2^ = 0.0%	Moderate
	4 weeks	-0.24 (-0.66 to 0.17)	—	Low
	8 weeks	-0.57 (-1.0 to -0.13)	—	Low
	12 weeks	-0.25 (-0.69 to 0.19)	—	Low
	16 weeks	0.17 (-0.52 to 0.85)	Q = 79.7, df = 3, I^2^ = 92.9%	High
	**Metoprolol**			
	Baseline	0.0 (-0.37 to 0.37)	—	Low
	8 weeks	0.0 (-0.38 to 0.38)	—	
	**Nadolol**			
	Baseline	0.00 (-0.67 to 0.84)	—	Low
	24 Weeks	0.86 (0.07 to 1.7)	—	
	**Naproxen**			
	12 weeks	0.14 (-0.29 to 0.56)	—	Low
	**Nimodipine**			
	24 weeks	-0.50 (-0.89 to -0.11)	—	Low
	**Propranolol (80 v 160mg doses)**			
	Baseline	0.00 (-0.40 to 0.40)	—	Low
	8 weeks	0.00 (-0.51 to 0.51)	—	
	12 weeks	0.00 (-0.43 to 0.43)	—	
	**Riboflavin**			
	Baseline	0.32 (-0.08 to 0.71)	—	Low
	4 weeks	0.33 (-0.07 to 0.72)	—	
	8 weeks	0.21 (-0.18 to 0.60)	—	
	12 weeks	0.42 (0.02 to 0.82)	—	
	24 weeks	0.11 (-0.29 to 0.50)	—	
	**Timolol**			
	Baseline	0.00 (-0.31 to 0.31)	—	Low
	12 weeks	0.14 (-0.17 to 0.45)	—	
	**Tolfenamic Acid**			
	Baseline	0.26 (-0.37 to 0.68)	—	Low
	12 weeks	0.15 (-0.63 to 0.93)	Q = 4.53, df = 1, I^2^ = 77.8%	Moderate
	**Topiramate**			
	Baseline	-0.44 (-0.95 to 0.07)	—	Low
	4 weeks	-0.19 (-0.69 to 0.32)	—	
	8 weeks	0.23 (-0.28 to 0.74)	—	
**Headache Duration**				
**Comparison**		**Weighted Mean Difference (95% CI)**	**Heterogeneity**	**Quality of evidence**
**Bisoprolol (5mg)**	**Bisoprolol (10 mg)**			
	Baseline	2.4 (-4.3 to 9.1)	—	Low
	12 weeks	-4.8 (-9.9 to 0.31)	—	
**Metoprolol**	**Acupuncture**			
	12 weeks	-2.4 (-6.5 to 1.7)	—	Low
	**Bisoprolol**			
	Baseline	0.0 (-4.9 to 4.9)	—	Low
	12 weeks	0.30 (-4.2 to 4.8)	—	
	**Clomipramine**			
	4 weeks	-2.8 (-4.4 to -1.2)	—	Low
	**Flunarizine**			
	Baseline	-1.3 (-3.7 to 1.1)	—	Low
	4 weeks	-3.2 (-5.7 to -0.69)	—	
	8 weeks	-3.3 (-5.9 to -0.66)	—	
	12 weeks	-2.4 (-4.9 to 0.11)	—	
	16 weeks	-0.40 (-3.2 to 2.4)	—	
	20 weeks	-1.3 (-4.1 to 1.5)	—	
	**Metoprolol + Fluoxetine**			
	Baseline	0.10 (-0.15 to 0.35)	—	Low
	12 weeks	-0.53 (-0.75 to -0.31)	—	
	**Nebivolol**			
	16 weeks	11 (-18.6 to 40.6)	—	Low
	**Nifedipine**			
	Baseline	1.0 (-14.3 to 16.3)	—	Low
	4 weeks	15.8 (0.49 to 31.1)	—	
	28 weeks	12.2 (0.66 to 23.7)	—	
**Pindolol (7.5 mg)**	**Pindolol (15 mg)**			
	Baseline	-0.70 (-7.9 to 6.6)	—	Low
	4 weeks	-0.80 (-8.2 to 6.5)	—	
**Propranolol**	**5-hydroxytryptophan**			
	Baseline	6.1 (-1.1 to 13.3)	—	Low
	16 weeks	3.6 (-3.7 to 10.9)	—	
	**Biofeedback**			
	Baseline	0.04 (-2.5 to 2.6)	—	Low
	24 weeks	0.33 (-1.3 to 2.0)	—	
	**Candesartan**			
	12 weeks	6.3 (-1.9 to 14.5)	—	Low
	**Cyclandelate**			
	Baseline	6.7 (-22.5 to 9.1)	—	Low
	4 weeks	-12.6 (-37.4 to 12.2)	—	
	8 weeks	6.6 (-18.2 to 31.4)	—	
	12 weeks	-4.5 (-26.6 to 17.6)	—	
	**Flunarizine**			
	Baseline	2.7 (-1.2 to 6.6)	—	Low
	4 weeks	-0.55 (-4.8 to 3.7)	Q = 0.06, df = 1, I^2^ = 0.0%	Moderate
	8 weeks	0.29 (-3.3 to 3.9)	Q = 0.08, df = 1, I^2^ = 0.0%	Moderate
	12 weeks	-0.21 (-3.3 to 2.9)	Q = 0.09, df = 1, I^2^ = 0.0%	Moderate
	16 weeks	1.4 (0.23 to 2.6)	Q = 4.33, df = 4, I^2^ = 7.6%	High
	**Mefenamic Acid**			
	12 weeks	7.0 (-27.3 to 41.3)	—	Low
	**Metoprolol**			
	Baseline	-24.0 (-40.3 to -7.7)	—	Low
	4 weeks	4.2 (-12.1 to 20.5)	—	
	16 weeks	0.0 (-12.3 to 12.3)	—	
	28 weeks	33.0 (6.3 to 59.7)	—	
	**Nadolo**l			
	Baseline	-8.1 (-11.4 to -4.8)	—	Low
	24 weeks	-19.5 (-31.8 to -7.1)	—	
	**Nifedipine**			
	Baseline	-1.0 (-16.3 to 14.3)	—	Low
	4 weeks	-15.8 (-31.1 to -0.49)	—	
	16 weeks	-12.2 (-23.7 to -0.66)	—	
	28 weeks	20.0 (0.97 to 39.0)	—	
	**Nimodipine**			
	24 weeks	-4.0 (-7.9 to -0.08)	—	Low
	**Propranolol (80 mg vs 160mg)**			
	Baseline	0.0 (-1.2 to 1.2)	—	Low
	8 weeks	-0.30 (-6.9 to 6.3)	—	
	12 weeks	0.20 (-1.8 to 2.2)	—	
	**Riboflavin**			
	Baseline	0.10 (-0.29 to 0.49)	—	Low
	4 weeks	0.10 (-0.31 to 0.51)	—	
	8 weeks	0.00 (-0.26 to 0.26)	—	
	12 weeks	-0.10 (-0.39 to 0.19)	—	
	24 weeks	0.30 (-0.06 to 6.6)	—	
	**Timolol**			
	Baseline	0.00 (-2.6 to 2.6)	—	Low
	12 weeks	-0.03 (-2.3 to 2.3)	—	
	**Tolfenamic Acid**			
	Baseline	6.4 (-21.9 to 34.7)	—	Low
	12 weeks	1.7 (-4.6 to 8.0)	Q = 0.05, df = 1, I^2^-0.0%	Moderate
	**Topiramate**			
	Baseline	-1.3 (-4.8 to 2.3)	—	Low
	4 weeks	-1.2 (-4.6 to 2.3)	—	
	8 weeks	1.0 (-1.9 to 4.0)	—	
	**Valproic Acid**			
	Baseline	-1.4 (-9.9 to 7.2)	—	Low
	10 weeks	-0.70 (-4.0 to 2.6)	—	

### Chronic migraine

There were four trials that evaluated beta-blockers for chronic migraine headaches, none were placebo controlled. (Table 6). Propranolol was compared to flunarizine [151], nortriptyline [76], valproic acid [105] and to the combination of propranolol and flunarizine [151]. In addition, a combination of propranolol and topiramate was compared to topiramate alone [129]. Propranolol was no better than valproic acid or flunarizine and the combinations (propranolol + topiramate and propranolol + flunarizine) was no better than topiramate and flunarizine alone (Table 6).

**Table 6:** Non-episodic trials.

Chronic Migraine					
Comparison	Outcome	Time point	Effect	Heterogeneity	Quality of Evidence
Propranolol v. Placebo	50% reduction in headache (RR)	42 weeks	2.0 (0.94 to 4.3)	—	Low
Propranolol v. Flunarizine	Headache Frequency (headaches/month)	Baseline	3.0 (0.79 to 5.2)	—	Low
		8 weeks	1.0 (-1.5 to 3.5)	—	
Propranolol + Flunarizine vs. Flunarizine	Headache Frequency (headaches/month)	Baseline	2.0 (-0.19 to 4.2)	—	Low
		8 weeks	3.0 (0.56 to 5.4)	—	
	50% reduction in headache (RR)	8 weeks	1.3 (0.97 to 1.6)	—	Low
Propranolol v. Nortriptyline	Headache Frequency (Headaches/month)	Baseline	-1.0 (-4.7 to 2.7)	—	Low
		8 weeks	-9.0 (-12.7 to -5.3)	—	
Propranolol v. Propranolol + Nortriptyline	Headache Frequency (Headaches/month)	Baseline	-4.0 (-7.8 to -0.24)	—	Low
		8 weeks	-7.0 (-10.8 to -3.3)	—	
Propranolol + Topiramate v. Topiramate	Headache Frequency (headaches/month)	Baseline	0.0 (-0.28 to 0.28)	—	Low
		12 weeks	-0.80 (-2.3 to 0.67)	—	
	Health Related Quality of Life (MIDAS)	Baseline	0.00 (-0.28 to 0.28)	—	Low
		12 weeks	0.09 (-0.26 to 0.44)	—	
	50% reduction in headache (RR)	12 weeks	1.05 (0.63 to 1.7)	—	Low
		24 weeks	1.1 (0.79 to 1.8)	—	
Propranolol v. Valproic Acid	Headache Frequency (headaches/month)	8 weeks	4.8 (0.27 to 9.2)	—	Low
**Chronic Tension-Type Headache**					
Pindolol + Amitriptyline v. Placebo	Headache Frequency (headaches/month)	Baseline	1.4 (-2.3 to 5.0)	—	Low
		4 weeks	7.8 (-13.9 to -1.5)	—	
		8 weeks	-11.6 (-16.8 to -5.5)	—	
	50% reduction in headache (RR)	8 weeks	3.8 (1.5 to 9.3)	—	Low
	Headache Severity (SMD)	Baseline	0.29 (-0.34 to 0.91)	—	Low
		8 weeks	-0.68 (-1.3 to -0.04)	—	
Pindolol + Amitriptyline v. Amitriptyline	Headache Frequency (headaches/month)	Baseline	1.6 (-2.2 to 5.3)	—	Low
		4 weeks	0.64 (-5.2 to 6.4)	—	
		8 weeks	-1.2 (-6.4 to 4.1)	—	
	50% reduction in headache (RR)	8 weeks	1.4 (0.87 to 2.2)	—	Low
	Headache Severity (SMD)	Baseline	3.7 (2.7 to 4.7)	— —	Low
		8 weeks	-0.05 (-0.65 to 0.56)	—	

### Tension-type headache

There was only one trial evaluating tension-type headache, comparing the combination of pindolol and amitriptyline to placebo and to amitriptyline alone [57]. The combination of pindolol and amitriptyline was more effective than placebo at reducing headache frequency at 4 and 8 weeks (Table 6) and in reducing headaches by at least 50% (RR: 3.8, 95% CI: 1.5–9.3), but equally effective with amitriptyline.

### Adverse events

Participants on beta-blockers were more likely to experience side effects than those on placebo (RR: 1.2, 95% CI: 1.0–1.4), though they were not more likely to withdraw (RR: 0.99, 95% CI: 0.83 to 1.2). Specific side effects more common with beta-blockers included dizziness (RR: 1.5, 95% CI: 1.0–2.3) and fatigue (RR: 1.5, 95% CI: 1.2–2.0). Depression, gastrointestinal problems, paresthesia’s and weight gain were not significantly different than placebo.

Propranolol was the only beta-blocker with sufficient numbers of studies to perform sensitivity analysis. There was no evidence of publication bias for propranolol’s reduction of headache frequency at 8 weeks (Egger’s p = 0.77) or at 12 weeks (p = 0.62). There was no evidence of an effect of quality (p = 0.97), age (p = 0.71), percent women (p = 0.28), percentage of dropouts (p = 0.55), dose (p = 0.61), intention to treat analysis (p = 0.35), concealed allocation (p = 0.38) or appropriateness of blinding (p = 0.98).

## Discussion

This review included one hundred and eight randomized controlled trials. Nearly all evaluated the efficacy of beta-blockers for episodic migraine headaches. Compared to placebo, propranolol was effective in reducing episodic migraine frequency. The effect began as early as four weeks. Migraine headache sufferers experienced an average reduction of 1.3 headaches/month; this translates to a reduction from 4.8 to 3.5 headaches a month. Subjects given propranolol were more likely to report at least 50% reduction in headaches and to reduce their consumption of analgesic medications. In addition to reducing the number of headaches, the residual headaches were less severe and shorter in duration compared to those receiving placebo. Outcomes from the propranolol comparisons to placebo were rated as high-quality evidence. In three trials, metoprolol also reduced headache frequency, though the reduction was less than 1 headache a month. Conclusions regarding the efficacy of other beta-blockers is less certain, as most were studied in just one trial each. Atenolol, bisoprolol and timolol had weak evidence of benefit. Acebutolol, alprenolol and nadolol appeared to be ineffective in migraine prophylaxis. This is unlikely to be due to properties of the beta-blockers. Propranolol is nonselective as is alprenolol and nadolol. Metoprolol, also effective is a ß-1 selective drug as is atenolol, bisoprolol and acebutolol. Given that acebutolol, alprenolol and nadolol were only studied in one trial each, it is possible that this may be either random variation in outcomes or a problem with the trials (such as dose or duration). The network analysis suggests that the benefit of beta-blocker may be a class effect.

The literature comparing beta-blockers to other modalities consisted mostly of single-trials with the exception of the comparison of propranolol to metoprolol (moderate quality, no difference) and to flunarizine (high quality, no difference). Flunarizine, not available in the United States, is well-established as effective in treating migraine headache. Universally, beta-blockers were associated with bradycardia and with lower average pulse rates than placebo trials. This is not surprising given their impact on chronotropy. Other side effects more common among participants taking beta-blockers included dizziness and fatigue, though subjects on beta-blockers were not more likely to withdraw from the studies.

While these conclusions are similar to previous reviews, this analysis is a unique contribution in many ways, first it included many more trials than previously reported, including Chinese trials that had previously not been included. Secondly, all beta-blockers were carefully parsed by type of headache (tension v. migraine, episodic v. chronic). Third, this study examined outcomes at the specific times reported, it is common for meta-analyses to pool trials at the last time point, regardless of whether there were significant differences in that time-point. Fourth, trial sequential analysis that demonstrated the adequacy of the current database for propranolol, suggesting that there is low likelihood of type 1 error in the conclusions. Fifth, the network meta-analysis didn’t show clear benefit of one beta-blocker over another, suggesting a class effect, though other beta-blockers have weaker evidence for benefit.

An important question, unanswered in this review, is how effective beta-blockers are compared to other commonly used prophylactic regimens. Propranolol, metoprolol and flunarizine appear to have similar efficacy. The other comparisons were all single-trial comparisons, making definitive conclusions impossible. In a previous review, tricyclic antidepressants resulted in a reduction in headache frequency for patients experiencing chronic migraines of 1.3 SMD, compared to placebo, a large effect [17]. In this study, there was only had one trial on chronic migraines, and the calculated SMD was 0.58, about half of the effect previously reported for TCAs. In a network meta-analysis of chronic migraines, tricyclic antidepressants were more effective than propranolol [17] but propranolol was similar in efficacy to antiepileptics and flunarizine, similar to findings in this study, though it is important to note that the majority of trials for beta-blockers are for episodic rather than chronic migraines. Definitive answer to this comparative effectiveness question requires additional studies that directly compare the different prophylactic modalities.

This review has several important limitations. First, while the quality of evidence for the comparison between propranolol and placebo was high, in general, the remaining comparisons were of low quality, consisting mostly of underpowered single randomized trials. While all the included trials were randomized, there were significant methodologic problems; combining poorly designed studies can lead to questionable results. It is important to note that most of the comparisons were graded as being of low-quality evidence because of the paucity of studies and concern about study problems. Secondly, studies were inconsistent in reporting outcomes, so even when there were more than one trial available, specific outcomes may only be provided by a single study. Moreover, there were significant problems with selective reporting of outcomes and many studies did not collect information on headache frequency, the measure preferred by the International Headache Society. Third, the number of studies available precluded sensitivity analyses, such as assessing for publication bias or exploring sources of heterogeneity. Fourth, beta-blockers have been studied almost exclusively in the management of episodic migraine headaches. Their benefit for chronic episodic or tension-type headaches is uncertain. Fifth, because of the paucity of trials for most beta-blockers the network analysis was underpowered to show differences between beta-blockers, Sixth, the average age of participants was 38, and mostly female. While this reflects the demographics of headache, it limits applicability to older adults.

## Conclusions

Propranolol is effective in reducing the burden of patients with episodic migraine headaches, reducing headaches from 5 to 3 headaches a month. This means that migraine sufferers given propranolol will have substantial residual headache burden. Propranolol reduces headaches by more than 50% as well as reducing the number of analgesic medication doses required. It also reduces the severity or duration of the headaches experienced. Propranolol and metoprolol exert similar effects and propranolol is as effective as flunarizine. The data for other beta-blockers and other comparisons are less clear.

## Supporting information

S1 Table Prisma checklist. (DOC)

S2 Table Search strategy. (DOCX)

S3 Table Quality ratings of included trials. (DOCX)
