# Supplementary material for: Beta-blockers for the prevention of headache in adults, a systematic review and meta-analysis
Source: PLoS One. 2019 Mar 20;14(3):e0212785. doi: 10.1371/journal.pone.0212785 (PMC6426199; doi:10.1371/journal.pone.0212785)
Supplement: S2 Table — (DOCX) [file pone.0212785.s002.docx]

**Online Table 1. Search strategies**

**CENTRAL (CRSO)**

MESH DESCRIPTOR Adrenergic beta-antagonists

((beta-blocker* or propranolol or bucindolol or carteolol or carvedilol or labetalol or nadolol or oxprenolol or penbutolol or pindolol or sotalol or timolol or acebutolol or atenolol or betaxolol or bisoprolol or celiprolol or esmolol or metoprolol or nebivolol)):TI,AB,KY

#1 OR #2

MESH DESCRIPTOR Headache

MESH DESCRIPTOR Headache Disorders EXPLODE ALL TREES

((tension near3 headache*)):TI,AB,KY

( (headache* or migrain* or cephalgi* or cephalalgi*)):TI,AB,KY

#4 OR #5 OR #6 OR #7

#3 AND #8

**MEDLINE & MEDLINE in Process (OVID)**

1. (randomized controlled trial [pt] or controlled clinical trial [pt] or randomized controlled trials [mh] or random allocation [mh] or double blind method [mh] or single-blind method [mh] or clinical trial [pt] or clinical trials [mh] or ("clinical trial" [tw]) or ((singl*[tw] or doubl*[tw] or trebl*[tw] or tripl[tw] AND (mask*[tw] or blind*[tw])) or (placebos[mh] OR placebo*[tw] or random*[tw] or research design[mh:noexp] OR comparative study [mh] or evaluation studies [mh] or follow-up studies [mh] or prospective studies [mh] or control* [tw] or prospectiv*[tw] or volunteer*[tw]) NOT (animals[mh] NOT humans[mh])
2. (headache or headache disorder or migrain* or headache* or cephalgi* or cephalalgi* or tension*)
3. "Adrenergic beta-Antagonists"[Mesh] OR beta-blocker OR propranolol OR bucindolol OR carteolol OR carvedilol OR labetalol OR nadolol OR oxprenolol OR penbutolol OR pindolol OR sotalol OR timolol OR acebutolol OR atenolol OR betaxolol OR bisoprolol OR celiprolol OR esmolol OR metoprolol OR nebivolol”
4. #1 AND #2 AND #3

**EMBASE (OVID)**

1 Adrenergic beta-antagonists/

2 (Adrenergic beta-Antagonists"[Mesh] OR beta-blocker OR propranolol OR bucindolol OR carteolol OR carvedilol OR labetalol OR nadolol OR oxprenolol OR penbutolol OR pindolol OR sotalol OR timolol OR acebutolol OR atenolol OR betaxolol OR bisoprolol OR celiprolol OR esmolol OR metoprolol OR nebivolol”).tw.

3 or/1-2

4 Headache/

5 exp Headache Disorders/

6 (tension adj3 headache$).tw.

7 (headache$ or migrain$ or cephalgi$ or cephalalgi$).tw.

8 or/4-7

9 3 and 8

10 random$.tw.

11 factorial$.tw.

12 crossover$.tw.

13 cross over$.tw.

14 drug therapy.fs.

15 randomly.ab.

16 trial.ab.

17 groups.ab.

18 10 or 11 or 12 or 13 or 14 or 15 or 16 or 17

19 exp animals/ not humans.sh.

20 18 not 19

21 9 and 20

**Web of Science (ISI)**

# 1 TOPIC: ((Adrenergic beta-Antagonists"[Mesh] OR beta-blocker OR propranolol OR bucindolol OR carteolol OR carvedilol OR labetalol OR nadolol OR oxprenolol OR penbutolol OR pindolol OR sotalol OR timolol OR acebutolol OR atenolol OR betaxolol OR bisoprolol OR celiprolol OR esmolol OR metoprolol OR nebivolol))

#2 TOPIC: (tension near/3 headache*)

#3 TOPIC: (headache* or migrain* or cephalgi* or cephalalgi*)

#4 TOPIC: #2 or #3

#5 TOPIC: #1 and #4

**CNKI, Wanfang and CQVIP**

（1）“偏头痛” AND “β受体阻滞剂”

（2）“偏头痛” AND （“**倍他乐克**” OR "**美托洛尔**"）

（3）“偏头痛” AND （“**阿替洛达**” OR "**氨酰心安**"）

（4）“偏头痛” AND （“**索他洛尔**” OR "施太可"）

（5）“偏头痛” AND （“心得安” OR "**普萘洛尔**"）

（6）“偏头痛” AND （“**卡维地洛**” OR " 达利全洛得"）

The final retrieval is  : #1 AND #2 AND #3 AND #4 AND #5 AND #6
