# Supplementary material for: Beta-blockers for the prevention of headache in adults, a systematic review and meta-analysis
Source: PLoS One. 2019 Mar 20;14(3):e0212785. doi: 10.1371/journal.pone.0212785 (PMC6426199; doi:10.1371/journal.pone.0212785)
Supplement: S3 Table — (DOCX) [file pone.0212785.s003.docx]

Online Table 1. Quality Assessment of Included Trials

| **Study, Year** | **Intention to Treat** | **Adherence assessed** | **Adequate Randomization** | **Concealed Allocation** | **Adequate Blinding** | **Incomplete Outcomes Addressed** | **Free of Selective Reporting** | **Free of “Other” Bias** | **Industry Sponsorship** | **JADAD Score** |
| --- | --- | --- | --- | --- | --- | --- | --- | --- | --- | --- |
| Agius 2013 | No | No | Yes | Unclear | Unclear | Yes | Yes | Unclear | No | 5 |
| Ahuja 1985 | No | No | Unclear | Unclear | Yes | Yes | Yes | Unclear | Yes | 4 |
| Albers 1989 | Yes | Unclear | Yes | Yes | No | No | Yes | Unclear | Yes | 4 |
| al-Qassab 1993 | No | Yes | Unclear | Unclear | Yes | No | Yes | Unclear | Yes | 6 |
| Anderson 1981 | Unclear | Unclear | No | No | Unclear | No | Yes | Unclear | Unclear | 4 |
| Andersson 1983 | No | No | No | No | No | Yes | Yes | Unclear | No | 6 |
| Ashtari 2008 | Yes | Yes | No | No | Yes | Yes | No | Unclear | No | 8 |
| Baldrati 1983 | Unclear | Unclear | No | Yes | Unclear | Unclear | No | Unclear | No | 3 |
| Behan 1980 | Unclear | Unclear | No | No | Unclear | Unclear | No | Unclear | No | 2 |
| Bonuso 1998 | Unclear | Unclear | No | No | Unclear | Unclear | Unclear | Unclear | No | 1 |
| Bordini 1997 | Unclear | Unclear | No | No | Yes | Unclear | No | Unclear | No | 6 |
| Borgesen 1974 | No | No | Unclear | Unclear | Unclear | Unclear | No | Unclear | No | 1 |
| Briggs 1979 | No | No | Unclear | Unclear | Unclear | Yes | No | Unclear | No | 1 |
| Carroll 1990 | Unclear | Unclear | No | No | Unclear | No | Unclear | Unclear | No | 4 |
| Chen 2009 | Yes | Unclear | Unclear | No | No | Yes | Yes | Unclear | No | 3 |
| Dahlof 1987 | Yes | No | Unclear | Unclear | Yes | No | Yes | Unclear | No | 5 |
| Diener 1996 | Yes | No | Unclear | Unclear | Unclear | Unclear | Yes | Unclear | Unclear | 4 |
| Diener 2001 | Unclear | Unclear | No | Yes | Unclear | No | Yes | Unclear | Yes | 4 |
| Diener 2002 | Yes | Yes | Yes | No | Yes | Yes | Yes | Unclear | Yes | 8 |
| Diener 2004 | Yes | No | Unclear | Unclear | Yes | Yes | Yes | No | Yes | 6 |
| Domingues 2009 | Unclear | Unclear | No | Yes | Yes | No | Yes | Unclear | Yes | 6 |
| Ekbom 1972 | No | No | Unclear | Unclear | Unclear | No | Yes | Unclear | Unclear | 2 |
| Ekbom 1975 | No | No | Unclear | Unclear | Unclear | No | No | Unclear | Unclear | 2 |
| Ekbom 1977 | No | No | Unclear | Unclear | Unclear | No | No | Unclear | Unclear | 3 |
| Formisano 1991 | Unclear | Unclear | No | No | Unclear | Unclear | Unclear | Unclear | Unclear | 2 |
| Forssman 1976 | No | No | Unclear | Unclear | Yes | Unclear | Unclear | Unclear | Unclear | 6 |
| Forssman 1983 | No | Unclear | Unclear | Unclear | Yes | Unclear | No | Unclear | Unclear | 3 |
| Freitag 1984 | No | Yes | Unclear | Unclear | Yes | Yes | Yes | Unclear | Unclear | 3 |
| Gawel 1992 | Unclear | Unclear | Unclear | Unclear | Yes | Yes | Unclear | Yes | Unclear | 6 |
| Gerber 1991 | Unclear | Unclear | No | No | Unclear | No | Unclear | Yes | Unclear | 3 |
| Gerber 1995 | Unclear | Unclear | Unclear | Unclear | Unclear | Unclear | Unclear | Unclear | Unclear | 3 |
| Gong, 2016 | Yes | Unclear | No | No | No | Yes | Unclear | Unclear | No | 3 |
| Grotemeyer 1987 | No | No | Unclear | Unclear | Unclear | No | No | Unclear | No | 3 |
| Grotemeyer 1988 | Unclear | Unclear | No | No | Unclear | No | No | Unclear | No | 3 |
| Grotemeyer 1990 | Unclear | Unclear | No | No | Yes | No | No | Unclear | No | 5 |
| Havanka-Kannianen 1988 | Unclear | Unclear | No | No | Yes | Unclear | Unclear | Unclear | Unclear | 3 |
| Hedman 1986 | Unclear | Unclear | Yes | No | Unclear | Yes | Unclear | Unclear | Unclear | 4 |
| Hesse 1994 | Unclear | Unclear | No | No | Unclear | Unclear | Unclear | Unclear | Unclear | 2 |
| Holdorff 1977 | No | No | Unclear | Unclear | Unclear | No | Yes | Yes | Unclear | 2 |
| Holroyd 2010 | Yes | Yes | Yes | Yes | Yes | Yes | Unclear | Yes | Unclear | 8 |
| Jin, 2001 | Yes | Unclear | Unclear | No | No | Yes | Unclear | Unclear | No | 2 |
| Johannsson 1987 | No | No | Unclear | Unclear | Unclear | Yes | Unclear | Unclear | Unclear | 4 |
| Johnson 1986 | No | No | Unclear | Yes | Yes | Yes | Unclear | Unclear | Yes | 5 |
| Kangasniemi 1983 | Unclear | Unclear | No | No | Unclear | Unclear | Unclear | Unclear | Unclear | 4 |
| Kangasnieme 1984 | Unclear | Unclear | Yes | No | Yes | Unclear | Yes | Unclear | Unclear | 6 |
| Kangasniemi 1987 | No | No | Unclear | Unclear | Yes | Unclear | Yes | Unclear | Unclear | 6 |
| Kaniecki 1997 | Unclear | Unclear | No | No | Yes | Yes | No | Unclear | Unclear | 2 |
| Kass 1980 | Unclear | Unclear | No | No | Unclear | Unclear | No | Unclear | Unclear | 4 |
| Kaushik 2005 | Yes | Yes | Yes | No | No | Yes | Yes | Unclear | Unclear | 4 |
| Ke, 2003 | Yes | Unclear | Unclear | No | No | Yes | Yes | Unclear | No | 2 |
| Kjaersgard 1994 | Unclear | Yes | No | Yes | Unclear | Yes | Yes | Yes | Unclear | 6 |
| Klapper 1994 | Unclear | Unclear | No | No | No | Yes | Unclear | Unclear | Unclear | 1 |
| Kozubski 1995 | Unclear | Unclear | Yes | No | No | No | Yes | Unclear | No | 1 |
| Kuritzky 1987 | No | Yes | Unclear | Unclear | Unclear | Unclear | Unclear | Unclear | Unclear | 1 |
| Langohr 1985 | No | No | No | No | Unclear | No | Yes | Unclear | No | 1 |
| Li, 2006 | Yes | Unclear | Unclear | No | Unclear | Yes | No | Unclear | No | 2 |
| Liu, 2016 | Yes | Unclear | Unclear | No | No | Yes | Yes | Unclear | No | 1 |
| Louis 1985 | Unclear | Unclear | No | No | Yes | No | Yes | Unclear | Unclear | 6 |
| Lucking 1988 | Unclear | Unclear | No | No | Unclear | No | No | Unclear | Unclear | 3 |
| Ludin 1989 | Unclear | Unclear | No | Yes | Yes | Yes | Yes | Unclear | No | 6 |
| Ma, 2011 | Yes | Unclear | Unclear | No | Unclear | Yes | Yes | Unclear | No | 4 |
| Maissen 1991 | Unclear | Unclear | No | No | Yes | No | No | Unclear | No | 6 |
| Malvea 1973 | No | No | Unclear | Unclear | Unclear | No | Unclear | Unclear | Yes | 2 |
| Mikkelsen 1986 | No | No | Unclear | Unclear | Yes | No | Unclear | Unclear | No | 4 |
| Nadelmann 1986 | Yes | No | Unclear | Unclear | Yes | No | Yes | Unclear | No | 4 |
| Nambiar 2011 | Yes | No | No | No | No | Yes | No | Unclear | No | 3 |
| Nanda 1978 | No | Yes | Unclear | Unclear | Yes | No | Yes | Unclear | No | 4 |
| Olerud 1986 | Unclear | Unclear | Yes | No | Yes | Yes | Yes | Unclear | No | 5 |
| Olsson 1984 | Unclear | Unclear | No | No | Yes | Yes | Yes | Unclear | No | 6 |
| Palferman 1983 | No | No | Unclear | Unclear | Yes | No | Yes | Unclear | No | 4 |
| Pita 1977 | Yes | No | Unclear | Unclear | Unclear | Yes | No | Unclear | Unclear | 3 |
| Pradalier 1989 | No | Yes | Unclear | Unclear | Yes | Yes | Yes | Unclear | No | 6 |
| Ryan 1982 | No | Yes | Unclear | Unclear | Yes | Yes | Yes | Unclear | No | 5 |
| Sargent 1985 | No | No | Unclear | Unclear | Unclear | Yes | Yes | Unclear | No | 4 |
| Schellenberg 2008 | Unclear | Unclear | Yes | Yes | Unclear | Yes | Yes | Unclear | Yes | 4 |
| Shimell 1990 | Yes | Unclear | No | Yes | Yes | Yes | Yes | Unclear | No | 6 |
| Silberstein 2012 | Yes | Yes | Yes | No | Yes | Yes | Yes | Unclear | No | 8 |
| Siniatchkin 2007 | No | Yes | Unclear | Unclear | Unclear | Unclear | Yes | Unclear | No | 3 |
| Sorensen 1991 | Yes | Yes | No | No | Yes | Yes | No | Unclear | Yes | 8 |
| Soyka 1990 | Unclear | Unclear | No | No | Yes | No | Yes | Unclear | No | 5 |
| Standnes 1982 | No | No | Unclear | Unclear | No | Yes | Yes | Unclear | Yes | 4 |
| Steiner 1988 | No | Yes | Unclear | Unclear | Yes | No | Yes | Unclear | Yes | 6 |
| Stellar 1984 | No | No | Yes | Unclear | Yes | Yes | Yes | Unclear | No | 8 |
| Stensrud 1976 | No | Yes | Unclear | Unclear | Yes | Yes | No | Unclear | Unclear | 3 |
| Stensrud 1980 | No | No | Unclear | Unclear | Yes | No | No | Unclear | Unclear | 5 |
| Stovner 2014 | Yes | Yes | Yes | Yes | Yes | Yes | Yes | Unclear | Yes | 8 |
| Tfelt-Hansen 1984 | No | No | Unclear | Unclear | Yes | Yes | No | Unclear | Unclear | 6 |
| van de Ven 1997 | Yes | Yes | Unclear | Unclear | Unclear | Yes | Yes | Yes | Yes | 4 |
| Vilming 1985 | Unclear | Unclear | No | No | Unclear | No | Unclear | Unclear | No | 3 |
| Weber 1971 | No | No | Unclear | Unclear | Unclear | No | No | Unclear | No | 2 |
| Wideroe 1974 | No | No | Unclear | Unclear | Unclear | No | No | Unclear | Yes | 1 |
| Worz 1992 | Unclear | Unclear | No | No | Unclear | No | Yes | Unclear | No | 2 |
| Yang 2006 | Yes | Unclear | Unclear | No | Unclear | Yes | No | Unclear | No | 3 |
| Yang 2016 | Yes | Unclear | Unclear | No | Unclear | Yes | Unclear | Unclear | No | 3 |
| Yuan 2005 | Yes | Unclear | Unclear | No | No | Yes | No | Unclear | No | 1 |
| Zhou 2015 | Yes | Unclear | No | No | No | Yes | Yes | Unclear | No | 3 |
| Zhu 2005 | Yes | Unclear | Unclear | No | No | Yes | Unclear | Unclear | No | 1 |
| Ziegler 1987 | No | Yes | No | No | No | Yes | Unclear | Unclear | No | 3 |
